# Review wetenschappelijk congres American Diabetes Association

**DOI:** 10.1007/s12467-022-1203-2

**Published:** 2022-08-29

**3-7 juni 2022**

Het 82e jaarcongres van de American Diabetes Association (ADA) was dit jaar een hybride bijeenkomst met zowel online als offline bijeenkomsten en vond plaats in New Orleans. Een verslag hiervan kun u lezen op de volgende pagina's.

## 1. Noninvasive Hypoglycemia Detection during Real Car Driving Using In-Vehicle Data 

Vera Lehmann, Thomas Zueger, Martin Maritsch, Michael Notter, Simon Schallmoser, Caterina Bérubé, Caroline Albrecht, Mathias Kraus, Stefan Feuerriegel, Elgar Fleisch, Tobias Kowatsch, Sophie N. Lagger, Markus Laimer, Felix Wortmann, Christoph Stettler ***Bern, Switzerland; Munich, Germany; Zurich, Switzerland; Nuremberg, Germany; St. Gallen, Switzerland***

### Background

To develop a non-invasive machine learning (ML) approach to detect hypoglycemia during real car driving based on driving (CAN), and eye and head motion (EHM) data.

### Methods

We logged CAN and EHM data in 21 subjects with type 1 diabetes (18 male, 41 ± yrs, A1c 6.8 ± 0.7 % [51 ± 7 mmol/mol]) during driving in eu- (EU) and hypoglycemia (< 3.0 mmol/L, hypo). Participants drove in a car (Volkswagen Touran) supervised by a driving instructor on a closed test-track. Using CAN and EHM data, we built ML models to predict the probability of the driver being in hypo. To make our approach applicable to different generations of cars, we present 3 ML models: first, a model combining CAN+EHM, representing the modern car with integrated camera. Second, a CAN model using driving data only, since modern cars are not generally equipped with EHM tracking. Third, anticipating that autonomous driving will limit the role of CAN data in the future, we tested a model solely based on EHM.

### Results

Mean BG in EU and hypo was 6.3 ± 0.8 mmol/L and 2.5 ± 0.5 mmol/L (p < 0.001), respectively. The model CAN+EHM achieved an area under the receiver operating characteristic curve of 0.88 ± 0.05, sensitivity of 0.70 ± 0.30, and specificity of 0.83 ± 0 in detecting hypo. Further results are in Fig. 1**.**

**Figuur 1.**
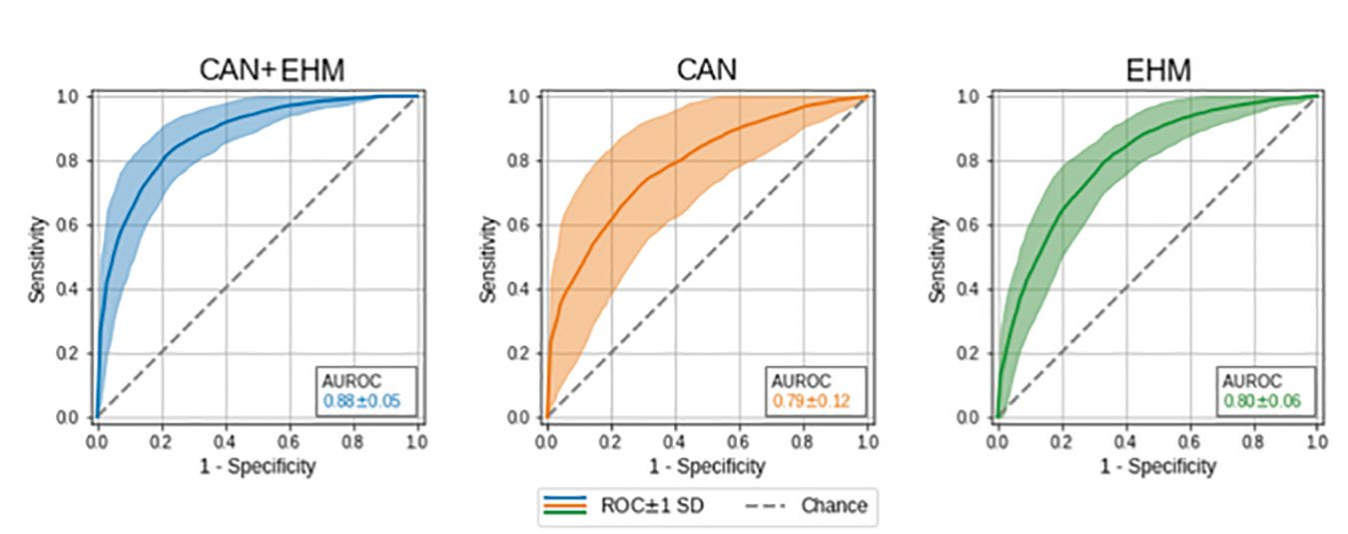
Machine learning detects hypoglycemia during real car driving based on driving (CAN) and eye and head motion (EHM) data. Shown is the area under the receiver operating characteristic curve (AUROC) for hypoglycemia detection using combined CAN+EHM data and CAN or EHM data, exclusively. ROC, receiver iperation characteristic; SD, standard deviation.

### Conclusion

We propose ML-based approaches to non-invasively detect hypo from driver behavior, applicable to contemporary cars and anticipating developments in automotive technology.

*Funding: Swiss National Science Foundation (SNF CRSII5_183569), Swiss Diabetes Foundation, Diabetes Center Berne, Automobile Club Switzerland (ACS), Federal Department of Defence, Civil Protection and Sport (DDPS) and Department of Research of the University Hospital Berne.*

### Commentaar

Technische innovaties hebben de behandeling van patiënten met diabetes drastisch verbeterd. Bij 'digitale diabetestechnologie' wordt meteen aan pompontwikkeling en sensorontwikkeling gedacht, maar op de ADA werd ook een hele andere vorm gepresenteerd. Een groep uit Zwitserland (ook weer eens wat anders) doet onderzoek naar de mogelijkheden van machinelearning (ML) ter ondersteuning van autorijdende patiënten met type 1-diabetes. Door het registreren van individuele gedragspatronen in bijvoorbeeld bewegen kan een kansrekening gegeven worden dat er een hypoglykemie is.

De techniek is nog in ontwikkeling. In deze studie werden 18 patiënten met type 1-diabetes gevolgd (HbA1c ≤ 70 mmol/l, leeftijd 21-60 jaar, met een Zwitsers rijbewijs). Zij deden een rit in een minivan, een Volkswagen Touran (onderzoeksgroep achterin de bak) op een buitenterrein van het Zwitserse leger. Hier en daar werd een obstakel toegevoegd (bijvoorbeeld een overstekende bergwandelaar). 

Vooraf aan de proef werd de bloedglucosewaarde met insuline verlaagd tot 2.0-2.5 mmol/l. Het gedrag en de verkeersgedragingen van de proefpersonen werden met een camera vastgelegd, waarbij de hoofdbewegingen apart werden vastgelegd. Het verschil tussen CAN, *eye-head movements* en de combinatie van de twee werden met elkaar vergeleken.

Analyse van de *Area Under the Receiver Operator Curve* (AUROC) liet zien dat de combinatie CAN en EHM het hoogste scoorde in de detectie van afwijkend gedrag, CAN en EHM individueel was iets minder goed, maar nog steeds bevredigend.

De conclusie is dat machinelearning met een waarschuwingsmogelijkheid een rol kan gaan spelen bij het nog veiliger maken van het autorijden voor mensen met diabetes. Daarbij wordt hier speciaal gedacht aan mensen met diabetes en *hypoglycemia unawareness*. Hierbij wordt ook gedacht aan het effect van hyperglykemie. Alexander Stork heeft belangrijk werk over diabetes en autorijden gedaan in de jaren 90 van de vorige eeuw; dit is een mooie follow-up.

*Review: Harold de Valk, internist-endocrinoloog, UMC Utrecht*

## 2. Symposium (Clinical Diabetes/Therapeutics) (zonder abstract) Complication compendium - Diagnosing and treating the overlooked offenders

T. Cukierman-Yaffe ***Tel-Aviv, Israel***

### Commentaar

In de zondagmiddagsessie: *Complication compendium - diagnosing and treating the overlooked offenders* werden een aantal minder bekende complicaties van diabetes besproken. Eén daarvan was cognitieve disfunctie en dementie. Gevreesde fenomenen voor mensen met en zonder diabetes. Het zwaartepunt van de voordracht lag bij type 2-diabetes.Een deel (prevalentie tot 20%, waarschijnlijk nog wel hoger) ontwikkelt een vorm van cognitieve disfunctie (vertraagde cerebrale processen), zonder grote verstoringen van de normale levensfuncties. Een deel van de groep met cognitief disfunctioneren gaat door naar dementie (vertraagde cerebrale processen), met ook functioneel verlies (disabilities). Cognitieve dysfunctie en dementie komt vaker voor bij type 2-diabetes. De odds ratio voor cognitief disfunctioneren is gemiddeld 1.5 in reviews van meta-analyses; dementie in het algemeen heeft ook een gemiddelde odds ratio van 1.5. De prevalentie van cognitief disfunctioneren kan oplopen tot wel boven de 20%. Bijdragende factoren zijn bijvoorbeeld vasculaire schade/lacunaire infarcten, hyperglykemie, slaapstoornissen, maar ook gehoorverlies wordt specifiek genoemd. Gehoorverlies als versterkende factor is een observationele bevinding; de causaliteit weten we niet. Maar het kan reden zij om het belang van (het dragen van) een gehoorapparaat te ondersteunen. Vasculaire schade (vasculaire dementie) heeft een odds ratio van 2.0. De invloed van slaapstoornissen is ook een observatie, die wel goed te plaatsen zou zijn. De disfunctie bij type 2- diabetes betreft met name de executieve functies, verwerkingssnelheid, geheugen en leermogelijkheden. In de praktijk van alledag kan zich dit manifesteren in langzaam of niet-adequaat reageren op hypoglykemie of fouten bij de dosis en injectietijd van insuline. Misschien kan een smart pen die aangeeft of insuline geïnjecteerd is, uitkomst bieden, al is het misschien maar voor een beperkte tijd.

*Review: Harold de Valk, internist-endocrinoloog, UMC Utrecht*

## 3. Need for Insulin Therapy in Gestational Diabetes Mellitus: A Predictive Model Assessment

Mariangela Caporusso, Ludovico Di Gioia, Gian Pio Sorice, Angelo Cignarelli, Annalisa Natalicchio, Francesco Giorgino, Luigi Laviola ***Rome, Bari, Italy***

### Background

A significant portion of pregnant women with Gestational Diabetes Mellitus (GDM) eventually require insulin therapy, thus necessitating closer monitoring. In this study, we assessed a predictive model for the need of insulin therapy in women with GDM. 

### Methods

In a retrospective cohort study, baseline data from 246 women with GDM (43% on subsequent insulin therapy, 57% on nutritional therapy alone) were analyzed using logistic regression. Diagnosis of GDM in previous pregnancies, previous GDM managed with insulin therapy, previous maternal impaired fasting glucose, fasting serum glucose diagnostic for GDM, and 0 h and 2 h diagnostic values at 75 g oral glucose tolerance test were independent qualitative significant predictors for subsequent insulin therapy. Pre-conceptional maternal body mass index, fasting serum glucose, HbA1c and gestational age at GDM diagnosis were independent quantitative significant predictors for subsequent insulin therapy.

### Results

According to the odds ratios produced by the logistic regression, a risk score was developed, with identification of low (score < 10, p < 0.001), moderate (score ≥ 10 and < 14, p = 0.052) and high (score ≥ 14, p < 0.001) risk categories for need of insulin therapy during pregnancy. The area under the ROC curve (AUC) for the internal validation of the predictive model was 0.724. The risk assessment tool was then validated with an independent cohort of 22 GDM women, resulting in a similar predictive power (AUC = 0.744).

**Figure:**
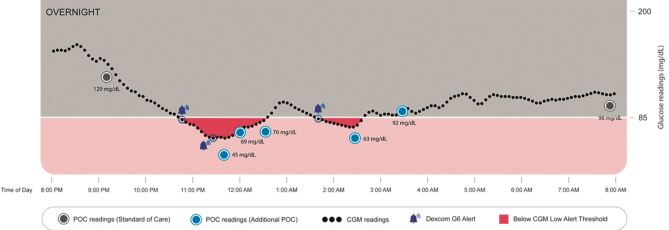

### Conclusion

Thus, a simple risk score based on easily available clinical and biochemical data at the first visit can predict the need for insulin therapy in GDM women, leading to closer monitoring and timely initiation of therapy in pregnancies at higher risk.

### Commentaar

Zwangerschapsdiabetes wordt frequent gevonden bij screening. Een deel van de patiënten zal tijdens de zwangerschap op insuline gezet moeten worden. Dit is een flinke domper voor de zwangere vrouw. Deze Italiaanse onderzoeksgroep heeft een studie gedaan om te zien of er een goed voorspellend model gemaakt kon worden om de kans op insulinebehandeling per persoon in te schatten en misschien het beleid daarop aan te passen. Zij voerden een retrospectieve studie uit, waarbij baseline data (bij de diagnose GDM) werden gescoord uit de status. Van de 245 vrouwen werd 43% later in de zwangerschap met insuline behandeld, 57% kon het alleen met een dieetadvies af. De volgende kwalitatieve factoren voorspelden een verhoogd risico op insulinebehandeling: eerdere zwangerschap met GDM, eerdere GDM met insulinebehandeling, ooit *impaired fasting glucose*, verhoogde nuchtere glucosewaarden, verhoogde glucosewaarden bij oGTT. Kwantitatieve factoren waren BMI voor zwangerschap, nuchter glucose, HbA1c en zwangerschapsduur bij diagnose. Deze lange lijst van factoren werd geanalyseerd in een logistisch regressiemodel in tertielen (laag, middel en hoog risico), resulterend in een risicoscore op insulinegebruik tijdens de zwangerschap. Deze leek in een kleine groep nieuwe patiënten met GDM een redelijk goede inschatting te geven (ROC AUC 0.742). Hoewel de Italiaanse poster wat rommeligs had, is het wel een sterk idee om de vrouwen met zwangerschapsdiabetes beter te informeren over de kans om insulinebehandeling nodig te hebben, en de begeleiding aan te passen. 

*Review: Harold de Valk, internist-endocrinoloog, UMC Utrecht*

## 4. Real-Time Continuous Glucose Monitoring in the Hospital Identifies and Prompts Treatment for Hypoglycemia

Matt Baker, John Welsh ***Kansas City, San Diego, USA***

### Background

Reliance on point-of-care (POC) blood glucose measurements for inpatient diabetes management can result in long between-measurement intervals, which contribute to undetected and untreated dysglycemia. 

### Methods

The G6 real-time continuous glucose monitoring (RT-CGM) system (Dexcom) allows for remote monitoring. In the hospital, this may reduce the need for close interactions and infectious disease transmission between patients and hospital staff. A man in his mid-60s was admitted to a non-intensive care unit in an acute care facility with a diagnosis of COVID-19. His preadmission type 2 diabetes was managed with twice-daily intermediate-acting insulin and prandial rapid-acting insulin; steroids and POC glucose testing (prandial and bedtime with a FreeStyle Precision Pro meter [Abbott]) were added to this regimen. A G6 was placed on the abdomen and was set to alert the staff to glucose values ≤ 85 mg/dL or predicted to be < 55 mg/dL within 20 minutes. 

### Results

Two hypoglycemic events occurred which triggered three G6 alerts (Figure). The G6 alerts prompted ad hoc POC measurements that confirmed the hypoglycemic events, prompted appropriate interventions, and documented the patient's return to euglycemia. All seven G6 readings were within 20 mg/dL or 20% of POC values ≤ 100 or > 100 mg/dL, respectively. 

### Conclusion

Inpatient RT-CGM use can detect existing or impending hypoglycemia and assist in diabetes management.

### Commentaar

Glucoseregulatie in het ziekenhuis blijft een pijnpunt. Vaak wordt gebruikgemaakt van metingen in bloed verkregen met de vingerprik, wat pijnlijk is voor de patiënt en logistieke problemen geeft voor de verpleegkundigen. Deze poster laat zien dat continue glucosemeting een goed alternatief kan zijn bij geselecteerde patiënten.

De auteurs presenteren een patiënt rond de 65 jaar die opgenomen wordt in een non-IC acute care unit vanwege COVID-19. Hij is bekend met type 2-diabetes, met tweemaal daags langwerkende insuline, naast kortwerkende insuline bij de maaltijden. Voor de COVID-19-behandeling werden steroïden gegeven. Hij kreeg een realtime continue glucosemeter (Dexcom G6) geplaatst, waarbij het hypo-alarm werd ingesteld op ≤ 4,7 mmol/l, dan wel een voorspelde waarde binnen 20 minuten van ≤ 3.0 mmol/l. Sensorwaarden werden doorgestuurd naar de smartphone van de patiënt en doorgeleid naar de tablet op de verpleegkundige post. Bij een lage waarde werd een vingerprik gedaan ter bevestiging.Tijdens de eerste dag van opname werd tweemaal een hypoglykemie geregistreerd: 2.5 mmol/l om 11 uur in de avond en eenmaal 3.5 mmol/l rond 3 uur in de nacht. Bij een normaal schema van vingerprikken waren deze hypoglykemieën niet gevonden. Deze ene casus illustreert een mogelijke toepassing in het ziekenhuis van de continue glucosemeting. Voordelen kunnen zijn: betere hypodetectie en minder verblijf bij of handelingen bij patiënten met een overdraagbare aandoening. En betere detectie van hypoglykemie (of hyperglykemische ontregeling) bij patiënten met diabetes die voor een andere aandoening in het ziekenhuis worden opgenomen. 

*Funding: Dexcom, Inc.*

*Review: Harold de Valk, internist-endocrinoloog, UMC Utrecht*

## 5. Hypoglycemia with Glimepiride vs. Insulin Glargine in the GRADE Study

Lawrence S. Phillips, Elizabeth R. Seaquist, Chelsea Baker, Richard M. Bergenstal, Nicole M. Butera, Jill P. Crandall, Robin Goland, Sophia H. Hox, Daniel S. Hsia, Mary L. Johnson, Erin Kazemi, Philip Raskin, Willy Valencia, Andrea H. Waltje, Naji Younes, Grade Research Group ***Decatur, Aurora, Bronx, New York, Honolulu, Baton Rouge, Minneapolis, Dallas, Charleston, Ann Arbor, Rockville, USA***

### Background

Hypoglycemia limits the glycemic control that can be achieved with insulin and sulfonylureas (SUs), but we lack evidence from head-to-head studies to guide management. 

### Methods

In the GRADE comparative effectiveness study, 5,047 patients with type 2 diabetes (T2DM) of < years' duration, on metformin monotherapy with HbA1c 6.8-8.5%, were randomized to addition of the SU glimepiride, insulin glargine U-100, sitagliptin, or liraglutide, permitting a direct comparison over 5.0 ± 1.3 (mean ± SD) years of follow-up. 

### Results

Glimepiride was initiated at 1-2 mg/day, glargine at 10-20 units/day, and both were titrated according to algorithms based on self-monitored blood glucose levels. Over 4 years, adjudicated severe hypoglycemia occurred in 2.3% of those randomized to glimepiride vs. 1.4% with glargine, but was less frequent with liraglutide (0.9%) and sitagliptin (0.7%), p = 0.003. During GRADE, HbA1c was measured every 3 months, and if a HbA1c > 7.5% was confirmed, "rescue treatment" with glargine and/or aspart was added. We examined management in participants who were unable to keep HbA1c ≤ 7.5% − whose primary study drug was insufficient − prior to their "rescue". At 3 months after randomization, hypoglycemic symptoms or a measured glucose < 70 mg/dl within the previous 30 days was reported by 33% of those using glimepiride vs. 15% with glargine (p < 0.001). The mean dose of glimepiride at 3 months (n = 627) and 12 months (n = 337) was 3.4 and 4.2 mg/day, respectively [a 24% increase but considerably submaximal (8 mg)]. In contrast, the dose of glargine at 3 months (n = 487) and 12 months (n = 337) was 26 and 37 units/day, respectively (a 44% increase, p < 0.0 vs. glimepiride). The outcome of a confirmed HbA1c > 7.5% was reached in 50% of those using glimepiride, vs. 39% with glargine (p < 0.001). 

### Conclusion

In metformin-treated patients with T2DM, there was more hypoglycemia, less increase in drug dose, and less preservation of glycemic control, with addition of the SU glimepiride compared to glargine; increases in glimepiride dose might have been limited by hypoglycemia.

*Funding: National Institute of Diabetes and Digestive and Kidney Diseases (U01DK098246, U34-DK-088043)*

**Gecombineerd commentaar op 5&6 onder abstract 6.**

## 6. Baseline OGTT as a Predictor of Type 2 Diabetes Progression in the GRADE Study

Kristina Utzschneider, Naji Younes, Nicole M. Butera, Ashok Balasubramanyam, Cyrus Desouza, Jonathan Krakoff, Joshua I. Barzilay, Ralph A. Defronzo, Richard M. Bergenstal, Tom A. Elasy, Willy Valencia, Charles Ma, Neda Rasouli, Steven E. Kahn, William Sivitz, Grade Research Group ***Rockville, Houston, Omaha, Phoenix, Duluth, San Antonio, Minneapolis, Brentwood, Charleston, Wilton, Denver, Seattle, Iowa City, USA***

Type 2 diabetes (T2D) is progressive, often requiring additional medications to maintain glycaemic control. We assessed baseline β-cell function and insulin sensitivity as predictors of worsening glycemia (HbA1c) in the GRADE Study. Adults with T2D < year and HbA1c 6.8-8.5% were randomized to glimepiride, sitagliptin, insulin glargine 100 U/mL or liraglutide added to metformin monotherapy. Complete baseline OGTT data were available for 4586 of 5047 participants (Mean ± SD: Age 57 ± year, 64% male, 5.0 ± 1.3 year f/up). Insulin sensitivity (HOMA2S) and early (C-peptide index (CPI): ΔCP/ΔG 0-30 min) and total (incAUC-CP/G 0-120 min) C-peptide responses were used in Cox proportional hazard models to predict time to 1° (HbA1c ≥ 7%) and 2° (HbA1c > 7.5%) glycaemic outcomes and tested for treatment interaction. Given the inverse relationship with insulin sensitivity, C-peptide responses were adjusted for HOMA2S. Values determined at baseline (mean ± SD) were CPI 0.8± 0. nmol/g, incAUC CP/G 1.0 ± 0.6 nmol/mg, HOMA2S 35 ± 68 %, fasting glucose 152 ± 31 mg/dl. Higher C-peptide responses predicted lower risk of 1° (CPI: HR ± SE per 1 unit change =0.74 ± 0.03, p < 0.001; incAUC-CP/G: HR = 0.68 ± 0.02, p < 0.001) and 2° glycaemic outcomes (CPI: HR = 0.71 ± 0.03, p < 0.001; incAUC-CP/G: HR = 0.60 ± 0.03, p < 0.001). Risks did not differ by treatment. There was interaction for HOMA2S and treatment; a 5 unit change in HOMA2S predicted lower risk of 1° and 2° outcomes with glimepiride and sitagliptin (HR = 0.97 ± 0. for both 1° outcomes; HR=0.96 ± 0. for 2° outcome with glimepiride, HR = 0.95 ± 0. for 2° outcome with sitagliptin; all p < 0.05) . A mg/dL change in fasting glucose predicted greater risk of 1° outcomes for all treatments, lowest for insulin glargine (HR=1.05 ± 0.01) and highest for sitagliptin (HR = 1.11 ± 0.01). Conclusions: Impaired β-cell function based on an OGTT predicted higher risk of T2D progression regardless of assigned treatment. This approach may identify cases that may benefit from earlier more aggressive therapy. 

*Funding: National Institute of Diabetes and Digestive and Kidney Diseases (U01DK098246, U34-DK-088043)*

### Commentaar 5&6

Type 2-diabetes is progressief en vereist vaak aanvullende medicijnen om de glykemische controle te behouden. Afgezien van zeer-hoogrisicopatiënten die reeds bekend zijn met hart- en vaatziekten en/of chronische nierschade is voor de meeste patiënten onduidelijk wat de optimale tweedelijnsbehandeling moet zijn na metformine. Er was tot nu toe echter geen uitgebreide vergelijking gemaakt van de langetermijneffecten en metabole effectiviteit van de veelgebruikte medicijnen voor de behandeling van deze aandoening. De GRADE-studie is gestart in 2013 om hier opheldering over te geven. Hieronder volgt een korte samenvatting van de belangrijkste bevindingen. Een post-hocstudie heeft vervolgens gekeken of β-celfunctie en/of de mate van aanwezige insulineresistentie, gemeten met een OGTT, voorspellers zijn voor therapiefalen.

### Methodes

Het onderzoek was een pragmatische gerandomiseerde, niet-geblindeerde klinische trial en omvatte 5047 deelnemers (gemiddelde leeftijd 57,2 jaar) uit de VS met minder dan tien jaar (mediaan 4,2 jaar) type 2-diabetes, HbA1c-niveaus van 51-69 mmol/mol (6,8-8,5%) en met een laag percentage deelnemers met reeds doorgemaakte hart- en vaatziekten (< 7%) die werden behandeld met metformine-monotherapie in een dosis van ten minste 500 mg/dag op het moment van screening. Metformine werd verhoogd tot een streefdosis van 2000 mg/dag zoals getolereerd tijdens een aanloopperiode van zes tot twaalf weken, met een minimale dosis van 1000 mg/dag bij aanvang van het onderzoek. Deelnemers werden vervolgens willekeurig toegewezen om de sulfonylureumderivaat glimepiride, de dipeptidylpeptidase-4-remmer sitagliptine, de GLP1-receptoragonist liraglutide of insuline glargine te ontvangen naast de voortgezette metforminebehandeling. De primaire en secundaire metabole eindpunten waren de tijd tot een bevestigde HbA1c ≥ 7% en > 7,5%, respectievelijk. Het initiëren van glargine in niet-glarginegroepen, en de tijd tot een HbA1c van > 7,5% na de initiatie van glargine was het tertiaire metabole eindpunt. Secundaire eindpunten waren microvasculaire uitkomsten, cardiovasculaire aandoeningen (CVD) en bijwerkingen.

### Resultaten

Het percentage deelnemers dat tijdens een gemiddelde follow-up van ongeveer vijf jaar HbA1c-spiegels boven 53 mmol/mol (7,0%) ontwikkelde − de primaire uitkomst − was het laagst in de glargine- en liraglutidegroepen (respectievelijk 67% en 68%), gevolgd door de glimepiride-arm (72%) en het hoogst in de sitagliptinegroep (77%). De gemiddelde tijd tot het primaire metabole eindpunt (dagen) was voor sitagliptine: 697, glimepiride: 810, glargine: 861 en liraglutide: 882. In paarsgewijze vergelijking hadden insuline glargine- en liraglutidegebruik een significant lager risico op de primaire uitkomst dan sitagliptine en glimepiride, terwijl sitagliptine zelfs een significant hoger risico hierop liet zien. In het eerste jaar waren liraglutide en glimepiride het effectiefst bij het verlagen van het HbA1c onder 53 mmol/mol (7,0%). Er waren geen significante verschillen voor MACE, Non-MACE en mortaliteit tussen de interventies. De percentages van alle cardiovasculaire (CV) aandoeningen samengenomen - gedefinieerd als MACE - ziekenhuisopname voor hartfalen, instabiele angina pectoris, voorbijgaande ischemische aanval of revascularisatie waren het laagst in de liraglutide- en glarginegroep (respectievelijk 5,8% en 7,6%), gevolgd door de groepen glimepiride (8,0%) en sitagliptine (8,6%). Liraglutide had een lagere cumulatieve incidentie (P-waarde = 0,048) in vergelijking met de andere interventies. De percentages van microvasculaire complicaties, waaronder nefropathie en distale sensorische polyneuropathie, waren vergelijkbaar in alle vier onderzoeksgroepen. In de veiligheidsanalyse waren de percentages ernstige bijwerkingen eveneens vergelijkbaar tussen de vier groepen, variërend van 33% met liraglutide tot 37% met glimepiride. Ernstige hypoglykemie kwam vaker voor bij deelnemers die met glimepiride werden behandeld (2,3%), in vergelijking met de andere behandelingen (0,7-1,4%). Gastro-intestinale klachten kwamen het meest voor bij liraglutide. De post-hoc studie toonde dat een verminderde β-celfunctie op basis van een OGTT een hoger risico op T2D-progressie voorspelde, ongeacht de gebruikte medicatie.

### Conclusie

De GRADE-studie is erin geslaagd om verschillende veelgebruikte interventies over een lange periode te vergelijken in patiënten met type 2-diabetes. Liraglutide en glargine bleken de effectiefste therapieën tijdens dit onderzoek. Secundaire resultaten gaven ook inzicht in een mogelijk bijkomend voordeel voor liraglutide: cardiovasculaire protectie. De gastro-intestinale bijwerkingen van liraglutide zijn duidelijk sterker in vergelijking met de andere. Echter bij een verminderde β-celfunctie bleek geen van de medicamenten in staat de progressie van de ziekte te stoppen. Een beperking van de studie is het niet kunnen evalueren van SGLT2-remmers, hetgeen de generaliseerbaarheid van de data beperkt. Ook geeft de studie weinig handvatten voor persoonsgerichte inzet van deze middelen. Misschien dat post-hocsubgroepanalyses daarbij nog behulpzaam gaan zijn.

*Review: Erik Serné, internist-vasculair geneeskundige, Amsterdam UMC*

## 7. Outcomes from the Insulin-Only Bionic Pancreas Pivotal Extension Study

Jane L. Lynch ***San Antonio, USA***

### Background

The bionic pancreas (BP) is initialized with body weight only, makes all insulin dosing decisions autonomously, and uses meal announcements without carbohydrate counting. After completing a Pivotal RCT, 90 of the 107 (aged 6-71) participants who were randomized to the 13-week baseline standard-care (SC) arm in the RCT participated in the 13-week Insulin-Only BP Pivotal Extension Study (ES). 

### Methods

Visit completion rate was 100%. During the baseline SC period, 1 adult participant had 2 severe hypoglycaemia (SH) events; the same participant had 2 additional SH events during the ES. During the baseline SC period there were no DKA events; 1 paediatric participant had a DKA event due to infusion set failure during the ES. A decrease of HbA1c, mean CGM glucose, percent of time < 70 mg/dl, percent of time > 180 mg/dl, and glucose coefficient of variability (CV), and increased percent of time in range (TIR) occurred in the Extension Study when compared to the baseline SC period. Changes from baseline were similar in adult (n = 42) and paediatric (n = 48) participants, except for reductions in time < 70 mg/dl and CV, which were statistically significant in the paediatric but not adult sub-groups (Table).

**Table:** Tabel 1.

**Metric**	**Pre-RCT Baseline**		**RCT**		**Transition Phase**	
	**BP** **Guidance**	**Pre-Study IR**	**BP** **Guidance**	**Pre-Study IR**	**BP** **Guidance**	**Pre-Study IR**
**MDI Users**	**(N=49)**	**(N=46)**	**(N=49)**	**(N=46)**	**(N=47)**	**(N=44)**
Mean glucose mg/dL *mean (SD)*	205 ± 45	196 ± 44	165 ± 14	162 ± 13	203 ± 41	198 ±4 4
% Time 70-180 mg/dL *mean (SD)*	41% ± 19%	46% ± 19%	65% ± 9%	66% ± 8%	42% ± 19%	44% ± 22%
% Time <54 mg/dL *median (quartiles)*	0.12% (0.03%, 0.45%)	0.23% (0.05%, 0.59%)	0.20% (0.09%, 0.47%)	0.30% (0.19%, 0.43%)	0.00% (0.00%, 0.00%)	0.00% (0.00%, 0.37%)
**Insulin Pump Users**	**(N=50)**	**(N=58)**	**(N=50)**	**(N=58)**	**(N=46)**	**(N=57)**
Mean glucose mg/dL *mean (SD)*	181 ± 38	181 ± 33	158 ± 11	160 ± 15	164 ± 27	174 ± 33
% Time 70-180 mg/dL *mean (SD)*	53% ± 17%	52% ± 17%	68% ± 7%	68% ± 11%	61% ± 15%	55% ± 19%
% Time <54 mg/dL *median (quartiles (SD)*	0.20% (0.03%, 0.76%)	0.35% (0.05%, 0.79%)	0.37% (0.14%, 0.72%)	0.32% (0.16%, 0.64%)	0.41% (0.00%, 1.14%)	0.14% (0.00%, 0.51%)
**AID Users**	**(N=49)**	**(N=43)**	**(N=49)**	**(N=43)**	**(N=46)**	**(N=39)**
Mean glucose mg/dL *mean (SD)*	170 ± 28	176 ± 27	159 ± 16	59 ± 11	168 ± 32	165 ± 24
% Time 70-180 mg/dL *mean (SD)*	62% ± 16%	59% ± 15%	68% ± 9%	69% ± 7%	61% ± 18%	63% ± 16%
% Time <54-180 *median (quartiles (SD)*	0.15% (0.00%, 0.43%)	0.10% (0.00%, 0.52%)	0.38% (0.19%, 0.60%)	0.32% (0.16%, 0.47%)	0.30% (0.00%, 1.23%)	0.00% (0.00%, 0.48%)

### Conclusion

The 90 participants who randomized to a 13-week baseline SC period and transitioned to the 13-week ES had statistically significant improvements in HbA1c and CGM outcomes following transition to the Insulin-Only BP without increases in hypoglycaemia.

*Funding: Funding from National Institute of Diabetes and Digestive and Kidney Diseases (grant #1UC4DK108612-01) Funding and bionic pancreas devices from Beta Bionics, Inc. Fast-acting insulin aspart and insulin aspart provided by Novo Nordisk Insulin lispro by Eli LillyBlood glucose meters and test strips (Contour Next One Blood Glucose Monitoring System) provided by Ascensia Diabetes Care, Basel, CH. Continuous glucose monitor sensors and transmitters were purchased from Dexcom, Inc. at a discounted price.*

### Commentaar

Op de eerste dag van de 82e wetenschappelijke sessies van de American Diabetes Association werden de resultaten van de iLet Bionic Pancreas-studie van Beta Bionics gepresenteerd. De studie maakt deel uit van de data die worden ingediend bij de FDA om te proberen goedkeuring te krijgen voor het apparaat zodat het beschikbaar kan worden gemaakt voor mensen met diabetes. Op dit moment is het systeem enkel beschikbaar voor onderzoek (zoals in een klinische studie). Het iLet Bionic Pancreas-systeem maakt gebruik van een insulinepomp met slang die ongeveer zo groot is als een creditcard en die het *automated insulin delivery* (AID)-algoritme, een Dexcom G6 en een smartphone of ander apparaat herbergt. Het wordt gedragen op dezelfde manier als andere AID-apparaten, waarbij de pomp op de buik wordt bevestigd en de Dexcom G6 op de achterkant van de bovenarm of op de buik wordt gedragen. Een van de dingen die de iLet Bionic Pancreas zo uniek maakt, is de minimale input die van de gebruiker wordt vereist. Het enige dat nodig is, is het gewicht van de gebruiker wanneer ze het apparaat voor het eerst starten en vervolgens aanpassingen voor de glucosestreefwaarde (normaal, lager of hoger) en het maaltijdtype (ontbijt, lunch of diner) en grootte (gebruikelijk voor mij, minder of meer). Alle insulinedosering wordt vervolgens bepaald door het algoritme van het apparaat met behulp van deze informatie.

### Resultaten

Het betrof een *multicenter randomized control trial* (RCT) dat werkzaamheids- en veiligheidseindpunten vergeleek van het iLet Bionic Pancreas (BP-)systeem versus Usual Care (UC) gedurende een onderzoeksperiode van 13 weken. Er waren 440 deelnemers (165 kinderen en tieners en 275 volwassenen). De studie was relatief divers, met bijna 1 op de 4 deelnemers die zich identificeerden als niet-blank. Aan het begin van de studie gebruikte 88% van de deelnemers al een continue glucosemonitor (CGM) en 31% gebruikte al een hybride closed-loop AID-systeem. De deelnemers werden willekeurig ingedeeld in een van de drie verschillende behandelingsgroepen: 1. Een groep bestaande uit zowel kinderen als volwassenen die hun standaard insulinetoedieningsmethode voortzetten (welke technologie ze in het begin ook gebruikten) met een Dexcom G6 CGM. 2. Een groep bestaande uit zowel kinderen als volwassenen die de kunstmatige alvleesklier gebruikten met Humalog en Novolog insuline. 3. Een groep van enkel volwassenen die de kunstmatige alvleesklier met Fiasp-insuline gebruikten. Ten opzichte van de standaard zorggroep met continue glucosemonitoring (CGM) werd een gemiddelde daling van HbA1c van 0,5% gezien op de iLet in elk van de bovengenoemde 3 groepen: 43% van de volwassen deelnemers aan de iLet met Humalog/NovoLog zag een vermindering van 0,5% of meer in HbA1c in vergelijking met slechts 17% van degenen in de standaardzorggroep. In verschillende subgroepen van volwassenen (ras/etniciteit, opleiding, inkomen en baseline insulinetherapieën) waren de 13-weekse HbA1c-resultaten consistent 7,0-7,3% op de iLet Bionic Pancreas met een sterkere verbetering bij een hoger baseline HbA1c. Bij jongeren (leeftijd 6-17 jaar) met een initieel HbA1c > 9%, zagen degenen in de kunstmatige alvleeskliergroep een toename in Time in Range (TIR) bereik van 31% in vergelijking met de standaardzorggroep; deze toename in tijd in bereik is gelijk aan een extra 7,4 uur/dag. 51% van de jongeren (leeftijd 6-17 jaar) op de iLet met Humalog/NovoLog zag een vermindering van 0,5% of meer in HbA1c vergeleken met slechts 8% in de standaardzorggroep. 29% van de jongeren (leeftijd 6-17 jaar) zag een HbA1c-verlaging van 1% of meer op de iLet met Humalog/NovoLog vergeleken met 6% van degenen in de standaardzorggroep. Volwassen gebruikers in de kunstmatige alvleeskliergroep ervoeren een vermindering van *diabetes distress* en angst voor hypoglykemie. Ouders van kinderen in de kunstmatige alvleeskliergroep rapporteerden een significante toename van de tevredenheid over diabetesbehandeling in vergelijking met de standaardzorggroep. In de volwassen groep die de kunstmatige alvleesklier met Fiasp gebruikte, waren de resultaten bijna identiek aan de groep die de kunstmatige alvleesklier met Humalog en Novolog gebruikte. Het enige grote verschil was een kleine toename van het aantal deelnemers dat een Time in Range van meer dan 70% bereikte (58% van de deelnemers in de kunstmatige alvleesklier met Fiasp-groep en slechts 47% in de kunstmatige alvleesklier met Humalog/Novolog-groep).

### Conclusie

De bovengenoemde resultaten zijn zeer indrukwekkend. De kunstmatige alvleesklier vertoonde significante verbeteringen in HbA1c en Time in Range, zelfs in vergelijking met mensen die al een hybride closed-loop AID-systeem zoals Control-IQ of MiniMed 670G gebruikten. Beta Bionics, de fabrikant van de bionisch pancreas, test momenteel ook het Bihormonal iLet Bionic Pancreas AID-systeem. De hoop is dat met toevoeging van glucagon glucosespiegels kunnen worden gestabiliseerd met nog minder interactie van de gebruiker, waarbij onder andere de noodzaak voor koolhydrateninvoer wordt geëlimineerd. In Nederland wordt op dit moment eveneens de bihormonale kunstmatige alvleesklier van INREDA getest.

*Review: Erik Serné, internist-vasculair geneeskundige, Amsterdam UMC*

## 8. ADA Presidents' Select Abstract: Effects of Tirzepatide vs. Insulin Glargine 100 U/mL on Kidney Outcomes in Participants with Type 2 Diabetes in SURPASS-4

Hiddo L. Heerspink, Naveed Sattar, Imre Pavo, Axel Haupt, Kevin L. Duffin, Zhengyu Yang, Russell Wiese, Katherine R. Tuttle, David Cherney ***Groningen, Netherlands; Glasgow, United Kingdom; Indianapolis, USA; Spokane, USA; Toronto, Canada***

### Background

Tirzepatide (TZP, 5, 10, 15 mg/week), a dual GIP/GLP-1 receptor agonist, reduced HbA1c levels more than titrated daily insulin glargine (iGlar) in people inadequately controlled on oral diabetes treatments with type 2 diabetes (T2D) and high cardiovascular risk (median study duration 85 weeks).

### Methods

Here, we compared progression to pre-specified kidney endpoints between TZP and iGlar. Composite kidney outcomes in SURPASS-4 were analysed: endpoint 1 (eGFR [CKD-EPI] decline ≥ 40% from baseline, renal death, progression to ESRD, new onset macroalbuminuria) and endpoint 2 (endpoint 1 without new onset macroalbuminuria).

### Results

Data were examined within the entire study population, and in subgroups defined by baseline SGLT2i use, UACR ≥ 30 mg/g, eGFR < 60 mL/min/1.73m^2^ and in those at high risk for kidney related outcomes, defined as eGFR < 75 mL/min per 1.73 m^2^ and macroalbuminuria, or eGFR < 45 mL/min per 1.73 m^2^. At baseline, participants (n = 1995, age 63.6 years, HbA1c, 8.5%) had a mean eGFR of 81.3 mL/min per 1.73 m^2^; 17% had eGFR < 60 mL/min per 1.73 m^2^, 28% microalbuminuria (UACR 30-300 mg/g) and 8% macroalbuminuria (UACR > 300 mg/g). During the follow-up to 1 weeks, TZP participants experienced significantly fewer renal outcomes, especially new onset of macroalbuminuria versus iGlar (Table).

**Tabel 2.** Kidney endpoints in pooled tirzepatide (5, 10, 15mg) and insulin glargine treatment arms of SURPASS-4 Data are from the mITT population (effecacy analysis set), including on treatment data prior to the use of rescue therapy. Cox proportional-hazards model was used to estimate the HR and 95% CI for pooled TZP compared with iGlar for the endpoints. HR estimate with CI is not calculated when either the TZP or iGlar arm has no event. aeGFR decline ≥40% from baseline, renal death, progression to ESRD, and new onset macroalbuminnuria. beGFR decline ≥40% from baseline, renal death, and progression to ESRD. ceGFR <60 CKD-EPI mL/min per 1.73 m2. deGFR <75 CKD-EPI mL/min per 1.73 m2 and macroalbuminnuria, or eGFR <45 CKD-EPI mL/min per 1.72 m2. TZP 5 mg, and 15 mg arms pooled for analysis. *P<.05 versus iGlar. CI=confidence interval; CKD-EPI=Chronic Kidney Disease Epidemiology Collaboration; ESRD=end stage renal disease; eGFR=estimated glomerular filtration rate; HR=hazard ratio; iGlar=insulin glargine; mITT=modified intention-to-treat; N=number patients in populationl; n=number of patient with eventl SGL T2i=spdium-glucose co-transporter 2 inhibitors; TZP=tizepatide; UACR=urine albumine-creatinine ratio.

		**Composite** **endpoit 1a**		**Composite** **endpoit 2b**		**eGFR decline ≥40% from baseline**		**Renal death**		**Progression to ESRD**		**New onset macroalbuminuria**	
**Population**	**Treatment**	**n** **(%)**	**HR** **(95% CI)**	**n** **(%)**	**HR** **(95% CI)**	**n** **(%)**	**HR** **(95% CI)**	**n** **(%)**	**HR** **(95% CI)**	**n** **(%)**	**HR** **(95% CI)**	**n** **(%)**	**HR** **(95% CI)**
**SURPASS-4** **population**	TZP N=995	64 (6.4)	**0.59** (0.43,0.80)*	39 (3.9)	0.80 (0.53,1.22)	38 (3.8)	0.86 (0.56,1.33)	1 (0.1)	0.99 (0.06,15.80)	0 (0.0)	-	25 (2.5)	0.41 (0.26,0.66)*
	iGlar N=1000	105 (10.5)		48 (4.8)		45 (4.5)		1 (0.1)		5 (0.5)		61 (6.1)	
**SGLT2i** **use at baseline**	TZP N=245	15 (6.1)	0.66 (0.34,1.26)	7 (2.9)	0.90 (0.33,2.47)	7 (2.9)	0.93 (0.34,2.55)	0 (0.0)	-	0 (0.0)	-	8 (3.3)	0.54 (0.23,1.27)
	iGlar N=256	23 (9.0)		8 (3.1)		8 (3.1)		0 (0.0)		1 (0.4)		15 (5.9)	
**No SGLT2i** **use at baseline**	TZP N=750	49 (6.5)	0.57 (0.40,0.81)*	32 (4.3)	0.78 (0.49,1.23)	31 (4.1)	0.85 (0.53,1.37)	1 (0.1)	0.98 (0.06,15.64)	0 (0.0)	-	17 (2.3)	0.37 (0.21,0.65)*
	iGlar N=744	82 (11.0)		40 (5.4)		37 (5.0)		1 (0.1)		4 (0.5)		46 (6.2)	
**Albuminuria ≥30 mg/g**	TZP N=358	35 (9.8)	0.47 (0.31,0.71)*	20 (5.6)	0.70 (0.39,1.25)	19 (5.3)	0.75 (0.41,1.37)	1 (0.3)	0.96 (1.06,15.31	0 (0.0)	-	15 (4.2)	0.33 (0.18,0.61)*
	iGlar N=349	65 (18.6)		27 (7.7)		24 (6.9)		1 (0.3)		3 (0.9)		39 (11.2)	
**Albuminuria ≤30 mg/g**	TZP N=621	27 (4.3)	0.70 (0.43,1.14)	19 (3.1)	0.92 (0.49,1.71)	19 (3.1)	0.97 (0.52,1.80)	0 (0.0)	-	0 (0.0)	-	8 (1.3)	0.42 (0.18,0.94)*
	iGlar N=630	39 (6.2)		21 (3.3)		21 (3.3)		0 (0.0)		0 (0.3)		21 (3.3)	
**Moderate or severely reduced kidney functionc**	TZP N=176	12 (6.8)	0.46 (0.23,0.93)*	5 (2.8)	0.37 (0.13,1.02)	4 (2.3)	0.40 (0.13,1.27)	1 (0.6)	-	0 (0.0)	-	7 (4.0)	0.68 (0.26,1.74)
	iGlar N=166	24 (14.5)		13 (7.8)		11 (6.6)		0 (0.0)		2 (1.2)		11 (6.6)	
**High risk for kidney related outcomesd**	TZP N=92	10 (10.9)	0.59 (0.27,1.29)	6 (6.5)	0.51 (0.19,1.35)	5 (5.4)	0.62 (0.21,1.84)	1 (1.1)	1.05 (0.07,16.85)	0 (0.0)	-	4 (4.3)	0.91 (0.24,3.38)
	iGlar N=94	17 (18.1)		12 (12.8)		9 (9.6)		1 (1.1)		2 (2.1)		5 (5.3)	

### Conclusion

In people with T2D and high cardiovascular risk, TZP reduced markers of diabetic kidney disease risk.

*Funding: Eli Lilly and Company*

### Commentaar

Afgelopen jaren hebben GLP-1RA's de behandeling van T2D getransformeerd door hun effectieve glucoseverlaging en bijkomend gewichtsverlies. Belangrijker, GLP-1RA's hebben bewezen gunstige effecten op mortaliteit, cardiovasculaire incidenten en progressie van diabetische nierziekte.^1^ De maximale dosering/werkzaamheid van GLP-1RA's kan worden beperkt door gastro-intestinale bijwerkingen. In een zoektocht naar verbeterde werkzaamheid worden begeleidende therapieën (lees: *aanvullend op *GLP-1R-agonisme) onderzocht. Het andere incretinehormoon, GIP, is op het eerste gezicht de natuurlijke partner van GLP-1, en is zelfs dominanter als insulinotroophormoon in de normale fysiologie. Tirzepatide, een unimoleculaire duale agonist van GLP-1- én GIP-receptoren, werd vanuit dit "twincretin-concept" ontwikkeld, en is recent door de FDA goedgekeurd als T2D-behandeling.^2^ Ruim 15 jaar na de introductie van GLP-1RA's en DPP-4-remmers, lijkt tirzepatide een nieuw tijdperk van nog *effectievere* incretinetherapieën in te luiden. In zijn fase-III-registratieprogramma SURPASS, verlaagde de maximaal aanbevolen dosering tirzepatide (15mg/week) het HbA1c met 1.5-2.0%, opvallend genoeg met 0.5%-punt *meer* dan GLP-1RA semaglutide (1mg/week) in een directe vergelijking.^3^Tirzepatide was ook effectiever dan semaglutide in het verlagen van lichaamsgewicht (ETD -5.5 kg) en bloeddruk, en het verbeteren van het lipidenprofiel.^3^

In deze renale-analyse van SURPASS-4 toont tirzepatide hoopgevende voordelen op nier-eindpunten in T2D patiënten met een hoog cardiovasculair risico. Het aandeel patiënten met chronische nierschade (CNS) was beperkt, en de follow-up *voor nefrologiebegrippen* kort ("slechts" mediaan 85 weken). Dit verklaart dat de incidentie van "harde" renale-eindpunten (waaronder eindstadium-nierfalen en renale dood) laag was. Dit gegeven rechtvaardigt - met voorzichtigheid - het gebruik van surrogaatuitkomsten, zoals (macro)albuminurie, eGFR-beloop, en ≥ 40%-afname in eGFR.^4^ Een interessante en relevante toevoeging in het studiedesign is het effect op albuminurie en eGFR na *staken* van de studiemedicatie (gemeten na 4 weken). Tirzepatide verlaagde de UACR en vertraagde eGFR-achteruitgang, terwijl de UACR juist sterk opliep in de iGlar-groep. Een *verhoogd* renaal risico met iGlar is onwaarschijnlijk op basis van bijvoorbeeld de ORIGIN-studie.^5^ Na staken van tirzepatide steeg de UACR weer, wat een potentieel behandeleffect onderstreept. Deze bevinding suggereert een renaal hemodynamisch tirzepatide-effect, zeker in combinatie met de ogenschijnlijke initiële "dip" in eGFR na *starten* van de therapie, en het eGFR-"herstel" richting uitgangswaarde na *staken*. 

Het voordeel op het 1^e^ gecombineerde renale eindpunt moet voorzichtig worden geïnterpreteerd, daar het gedreven wordt door verbetering in surrogaatuitkomst macroalbuminurie, maar de *hazard ratio* puntschattingen en CI-intervallen van het 2^e^ gecombineerde renale eindpunt (*zonder* macroalbuminurie) zijn veelbelovend. De onderzoekers sloten interactie met SGLT2-remmers uit, een elegante analyse die vaker zal moeten plaatsvinden nu deze geneesmiddelen (naast RAAS-remmers) als hoeksteen van CNS-behandeling moet worden beschouwd. Als laatste aandacht voor het HbA1c. Omdat glucoseverlaging *op zich* het risico op nierschade verlaagt, dient men bij renale analyses van studies met glucoseverlagende medicatie rekening te houden met HbA1c-verschillen tussen de groepen. Head-to-head studiedesigns als in SURPASS-4 - met iGlar als actieve controlegroep - zijn uitermate geschikt om dit verschil beperkt te houden. Echter, de opvallende sterkte glucoseverlagende effectiviteit van tirzepatide leidde in SURPASS-4 tot maar liefst 1.0% HbA1c-verschil tussen de groepen. Hoewel relevant, en hoewel de onderzoekers in mediatie-analyses hiervoor (nog) niet hebben gecorrigeerd, is het onwaarschijnlijk dat het renoprotectieve effect hier volledig door wordt verklaard.6 Ik zie uit naar de (renale) resultaten van uitkomststudies, waaronder de lopende veiligheidsstudie SURPASS-CVOT (NCT04255433; tirzepatide versus GLP-1RA dulaglutide), alsmede naar mechanistische studies die de niereffecten van tirzepatide (en de rol van GIP-receptoragonisme hierin) kunnen helpen verklaren.

#### Referenties

1. Sattar N, Lee MMY, Kristensen SL, et al. Cardiovascular, mortality, and kidney outcomes with GLP-1 receptor agonists in patients with type 2 diabetes: a systematic review and meta-analysis of randomised trials. Lancet Diabetes Endocrinol 2021;9(10):653-662.
2. https://www.fda.gov/news-events/press-announcements/fda-approves-novel-dual-targeted-treatment-type-2-diabetes
3. Frías JP, Davies MJ, Rosenstock J, et al. Tirzepatide versus Semaglutide Once Weekly in Patients with Type 2 Diabetes. N Engl J Med 2021;385(6):503-515.
4. Levin A, Agarwal R, Herrington WG, et al. International consensus definitions of clinical trial outcomes for kidney failure: 2020. Kidney Int 2020;98(4):849-859.
5. ORIGIN trial investigators. Basal insulin glargine and microvascular outcomes in dysglycaemic individuals: results of the Outcome Reduction with an Initial Glargine Intervention (ORIGIN) trial. Diabetologia 2014;57(7):1325-31.
6. Muskiet MHA, Tonneijck L, Smits MM, et al. GLP-1 and the kidney: from physiology to pharmacology and outcomes in diabetes. Nat Rev Nephrol 2017;13(10):605-628.

*Review: Marcel Muskiet, arts in opleiding tot internist - fellow endocrinologie en vasculaire geneeskunde, Amsterdam UMC*

## 9. The Renal and Vascular Effects of Combined SGLT2 and Angiotensin Converting Enzyme Inhibition 

Yuliya Lytvyn, Karen Kimura, Nuala Peter, Vesta S. Lai, Josephine Tse, Leslie Cham, Bruce A. Perkins, Nima Soleymanlou, David Cherney ***Burlington, Canada; Biberach, Germany; Toronto, Canada; Ridgefield, USA***

### Background

The goal of this mechanistic trial was to determine the kidney and cardiovascular effects of combined treatment with an SGLT2i (empagliflozin 25mg QD) and an ACEi (ramipril 10mg QD) for 4 weeks in patients at risk of renal hyperfiltration.

### Methods

In this randomized, double-blind, placebo-controlled, cross-over trial, measurements were obtained following each of the 4 treatment phases: 1) no treatment, 2) 4-week ramipril treatment alone, 3) 4-week empagliflozin-ramipril combination treatment, and 4) 4-week placebo-ramipril combination treatment. The primary endpoint was GFR after empagliflozin-ramipril treatment compared to placebo-ramipril. At the end of each study phase the following measurements were performed under clamped euglycemia (4-6 mmol/L): inulin (GFR) and para-aminohippurate (effective renal plasma flow, ERPF) clearances, tubular sodium handling, ambulatory blood pressure, arterial stiffness, heart rate variability, non-invasive cardiac output monitoring, plasma and urine biochemistry.

### Results

Empagliflozin-ramipril treatment resulted in an 8ml/min/1.73m^2^ lower GFR (p = 0.0061), lower absolute proximal fluid reabsorption rate (p = 0.0092), lower absolute proximal sodium reabsorption rate (p = 0.0056), and lower urinary 8-isoprostane (p = 0.0084) relative to placebo-ramipril treatment. Empagliflozin-ramipril treatment resulted in additive blood pressure lowering effects (SBP p = 0.0112; DBP p = 0.0032) and a lower total peripheral resistance (p = 0.0368). There were no other significant changes observed with the addition of empagliflozin.

### Conclusion

SGLT2i-ACEi combination treatment resulted in an expected GFR "dip", suppression of oxidative stress markers, and additive declines in blood pressure and total peripheral resistance in this mechanistic study. These changes are consistent with a protective physiological profile characterized by the lowering of intraglomerular pressure and related cardiorenal risk when adding an SGLT2i to conservative therapy.

*Funding: Boehringer Ingelheim and CIHR, Diabetes Canada and the Heart and Stroke Richard Lewar Centre of Excellence and the Heart and Stroke Foundation of Canada*

### Commentaar

De realisatie dat SGLT2-remmers beschouwd moeten worden als basisbehandeling voor patiënten met chronische nierschade (CNS) en hartfalen, ongeacht diabetesstatus, kent een mooie historie die aan elkaar hangt van toevalligheden. Initieel geïntroduceerd als glucoseverlagend medicijn voor T2D behandeling in 2012, nam het "verhaal" van SGLT2-remmers in 2015 een wending na de onverwachte voordelen van empagliflozine op MACE, hartfalen en nierschade in EMPA-REG OUTCOME.^1^ Sindsdien wordt het als cardiorenaal medicijn beschouwd, en hebben andere cardiovasculaireveiligheidsstudies in T2D, en specifieke studies in CNS- en hartfalenpatiënten, de voordelen van SGLT2-remmers ondersteund en uitgebreid. De voordelen zijn consistent in veel subgroepen, inclusief die mét en zónder T2D, verschillende stadia van nierschade, en hartfalen met behouden of verminderde ejectiefractie.^2^

Verschillende mechanistische verklaringen voor de cardiorenale effecten van SGLT2-remmers zijn opgeworpen. Centraal in de renoprotectieve verklaring staat het verlagen van glomerulaire hypertensie en hyperfiltratie via tubuloglomerulaire feedback.^3^ SGLT2-remmers blokkeren naast glucose ook natriumreabsorptie in de proximale tubulus, waardoor "stroomafwaarts" het natriumaanbod aan de macula densa toeneemt. De macula densa verlaagt vervolgens de glomerulaire druk via vasoconstrictie van afferente arteriolen, en vasodilatatie van efferente arteriolen.^3^ Hiermee reduceren SGLT2-remmers de fysieke belasting op de filtratiebarrière, albuminurie en zuurstofbehoefte voor tubulaire reabsorptie, en zijn mede daarom renoprotectief. Verschil tussen patiënten met T1D en T2D in de renaal-hemodynamische respons op SGLT2-remmers wordt gesuggereerd; in hyperfiltrerende T1D-patienten trad afferente vasoconstrictie op^4^, terwijl er in T2D-patienten vooral efferente vasodilatatie werd gezien.^5^ In de klinische praktijk zien we dit renaal-hemodynamische effect terug als initiële "dip" in eGFR na het starten van de SGLT2-remmer, die reversibel is nadat het medicijn wordt gestaakt. Deze "dip" kennen we ook bij het gebruik van RAAS-remmers; ACE-remmers en ARB's verlagen de glomerulaire druk door de effecten van angiotensine-II op de nier te remmen, leidend tot efferente vasodilatatie.^3^

Patiënten die op dit moment in aanmerking komen voor SGLT2-remmers in de klinische praktijk hebben vaak comorbiditeiten, zoals hypertensie en albuminurie. Vaak gebruiken ze dus ook andere medicijnen die op zichzelf van invloed kunnen zijn op renale hemodynamiek, zoals de RAAS-remmers, maar ook calciumkanaalblokkers, diuretica en NSAID's. Deze medicijnen kunnen daarmee de effectiviteit van SGLT2-remmers potentieel positief of negatief beïnvloeden. In deze elegante mechanistische studie werd de potentiële interactie tussen empagliflozine en ramipril onderzocht. De hypothese was dat de SGLT2-remmer in patiënten met hyperfiltratie vooral afferente vasoconstrictie zou veroorzaken, en de ACE-remmer vooral efferente vasodilatatie, met derhalve een potentieel additief effect op verlaging van de glomerulaire druk bij het gebruik van de combinatie. Inderdaad verlaagde empagliflozine de GFR in de aanwezigheid van ramipril, wat een (op lange termijn) renoprotectief effect van combinatietherapie ondersteund. Door een trage rekruteringssnelheid werden weinig patiënten met baseline hyperfiltratie geïncludeerd, waardoor helaas de hypothese en het mechanisme van GFR-daling (lees: afferent versus efferent) niet gedetailleerd kon worden onderzocht. Desalniettemin, de klinische waarde van de studie blijft overeind. Ook stipt deze studie een belangrijk aandachtspunt aan voor de toekomst: hoe kunnen we - vanuit onze kennis van de werkingsmechanismen van onze geneesmiddelen - de SGLT2-remmers het effectiefst gaan inzetten in de klinische praktijk? Ik pleit voor het uitvoeren van meer van dit soort mechanistische studies, waarin specifieke geneesmiddeleninteracties onderzocht worden die relevant kunnen zijn voor de praktijk.

### Referenties

1. Zinman B, Inzucchi SE, Lachin JM, et al. Empagliflozin, Cardiovascular Outcomes, and Mortality in Type 2 Diabetes. N Engl J Med 2015;373(22):2117-28.
2. Van der Aart-van der Beek AB, de Boer RA, Heerspink HJL. Kidney and heart failure outcomes associated with SGLT2 inhibitor use. Nat Rev Nephrol 2022;18(5):294-306.
3. Tonneijck L, Muskiet MHA, Smits MM, van Bommel EJ, Heerspink HJ, et al. Glomerular Hyperfiltration in Diabetes: Mechanisms, Clinical Significance, and Treatment. J Am Soc Nephrol 2017;28(4):1023-1039.
4. Cherney DZ, Perkins BA, Soleymanlou N, et al. Renal hemodynamic effect of sodium-glucose cotransporter 2 inhibition in patients with type 1 diabetes mellitus. Circulation 2014;129(5):587-97.
5. Van Bommel EJM, Muskiet MHA, van Baar MJB, et al. The renal hemodynamic effects of the SGLT2 inhibitor dapagliflozin are caused by post-glomerular vasodilatation rather than pre-glomerular vasoconstriction in metformin-treated patients with type 2 diabetes in the randomized, double-blind RED trial. Kidney Int 2020;97(1):202-212.

*Review: Marcel Muskiet, arts in opleiding tot internist - fellow endocrinologie en vasculaire geneeskunde, Amsterdam UMC*

## 10. Hypoglycaemia Frequency and Physiological Response to Double or Triple Doses of Once-Weekly Insulin Icodec vs. Once-Daily Insulin Glargine in T2D

Eva Svehlikova, Kristine Niss Arfelt, Roman Cailleteau, Sigrid Deller, Karen Margrete Thomsen, Marlies Hart, Ines Mursic, Thomas Pieber, Hanne Haahr ***Aalborg, Denmark; Graz, Austria; Søborg, Denmark***

### Background

Insulin icodec is a basal insulin in development for once-weekly (OW) dosing. The aim of this study was to compare the hypoglycaemia frequency and response after icodec vs. insulin glargine U100 (IGlar) overdosing.

### Methods

In a randomized, open-label, two-period crossover trial, 43 individuals with T2D on basal insulin ± metformin (mean ± SD age 56 ± 9 years, HbA1c 7.2 ± 0.7%) received OW icodec for 6 weeks and once-daily IGlar for 12 days at equimolar total weekly doses based on the individual daily run-in IGlar dose (mean 30 ± 14 U) titrated to a fasting SMPG target of 80-130 mg/dL. Once at steady state, double (DD) and triple (TD) doses of icodec and IGlar were followed by hypoglycaemia induction 44 hour (icodec) or 7 hour (IGlar) post-dose (expected time of maximum glucose-lowering effect): First, euglycemia was maintained at 100 mg/dL by variable i.v. glucose. Then, PG was allowed to decrease to a nadir of no less than 45 mg/dL maintained for 15 min. Euglycemia was restored by constant i.v. glucose. Hypoglycaemic symptom score (HSS) and counterregulatory hormones were assessed at PG 100 mg/dL and at predefined PG levels until nadir PG. 

### Results

For DD, clinically significant hypoglycemia (PG < 54 mg/dL) occurred in 40 vs. 36% of subjects for icodec vs. IGlar (odds ratio 1.28; p = 0.63). For TD, clinically significant hypoglycaemia occurred in 53 vs. 70% of subjects (odds ratio 0.48; p = 0.14), mean nadir PG was 56 vs. 52 mg/dL (treatment ratio 1.07; p < 0.001), change in HSS at nadir PG was comparable for icodec vs. IGlar (treatment difference 0.46; p = 0.77), responses in adrenaline, noradrenaline and cortisol during hypoglycaemia development were greater for icodec vs. IGlar, while glucagon and growth hormone levels increased similarly.

### Conclusion

A DD or TD of once-weekly insulin icodec does not lead to increased risk of hypoglycaemia compared to once-daily IGlar. During hypoglycaemia, a comparable symptomatic response and a moderately greater endocrine response were seen for icodec vs. IGlar.

*Funding: Novo Nordisk*

### Commentaar

NPH was lange tijd de meest gebruikte "langwerkende" insuline, met een werkingsduur van 12-18 uur. Klinisch heeft NPH nadelen: 1) een piekeffect dat het risico op hypoglykemie verhoogt, 2) onvoldoende activiteitsduur om nachtelijke/prandiale fasen te dekken, met risico op hyperglykemie, en 3) variabiliteit in effectiviteit van dag-tot-dag en patiënt-tot-patiënt. Onderzoek richtte zich afgelopen decennia op langdurigere, pieklozere en beter reproduceerbare insulines. De basale insulinetherapie is geëvolueerd van analogen van eerste generatie (glargine U-100, detemir) naar analogen van de tweede generatie (degludec, glargine U-300). Elke ontdekking was een stap dichter bij het doel: nabootsen van de continue insulinesecretie door bètacellen tussen maaltijden, tijdens perioden van vasten en 's nachts. Nieuwe generaties insulines hebben de kwaliteit van leven van diabetespatiënten verbeterd. Daar waar steeds sneller werkende prandiale insulines kunnen worden toegediend vlak vóór of - indien nodig - direct ná een maaltijd^1^, kunnen langwerkende basale insuline-analogen worden gegeven met tijdsintervallen variërend van 8-40 uur zonder afbreuk te doen aan de glykemische controle of veiligheid.^2^

Huidig onderzoek richt zich op nog langer werkende basale insuline-analogen: eenmaal per week. De gedachte is dat (voornamelijk T2D-)patiënten verlaging van de doseringsfrequentie van injecteerbare therapieën zullen waarderen. Een eenvoudiger en minder intensief toedieningsschema (van 365 naar 52 injecties/jaar) kan de drempel tot het gebruik ervan verlagen bij zowel de patiënt als diens behandelaar, en de kwaliteit van leven en therapietrouw verbeteren. Op hun beurt leidt dit tot hypothetisch snellere en betere glykemische controle. Na enkele gestrande pogingen zijn momenteel twee wekelijkse insulines in ontwikkeling: insuline icodec en 'basale insuline Fc'. Icodec is in het heden het uitgebreidst onderzocht. Structurele eigenschappen van icodec zorgen voor sterke reversibele binding aan albumine (~10x die van detemir), lagere affiniteit voor insulinereceptoren, en verminderde receptorgemedieerde klaring en enzymatische afbraak. De eerste fase-III-resultaten met icodec zijn recent aangekondigd^3^, en lijken bemoedigend qua effectiviteit en veiligheid ten opzichte van glargine U-100 en degludec in voornamelijk T2D.

Hoewel wekelijkse insuline vooruitgang kan gaan betekenen, kunnen huidige praktische aspecten van de eenmaal daags behandeling niet zomaar worden geëxtrapoleerd. Er zullen nieuwe titratiestrategieën moeten worden ontwikkeld. Snelle glucoseverlaging wordt niet verwacht omdat *steady-state* ~3-4 weken duurt. Oplaaddoses zullen dus nodig zijn bij de start van behandeling, of wanneer wordt overgeschakeld van een eenmaal daags naar een eenmaal per week basale-insuline.^4^ Frequente aanpassingen van de insulinedosering zijn misschien niet mogelijk. De vraag is daarbij welke patiëntencategorie het geschiktst is voor de insulines eenmaal per week. De relatieve inflexibiliteit suggereert dat dit vooral T2D-patiënten zijn met stabiele glucoseregulatie, relatief lage variatie in basale insulinebehoeften (waardoor langzame, maar consistente wekelijkse titratie mogelijk is) en voorspelbare levensstijlen. Het gebruik bij T1D lijkt uitdagender, met name bij zeer fysiek actieve patiënten, maar kent de interessante mogelijkheid om de frequentie van diabetische ketoacidose te verminderen.

Doch, klinisch het belangrijkste vraagstuk blijft: moeten we ons zorgen maken over hypoglykemieën met deze middelen, met betrekking tot frequentie, ernst en duur (met misschien langzamer herstel)? Huidige data wijst erop dat het risico op hypoglykemieën van niveau 2 of 3 met insuline eenmaal per week relatief laag zijn, en niet groter dan geassocieerd met eenmaal daagse basale insuline.^5^ Bovendien werden geen episoden vastgesteld die niet reageerden op standaard corrigerende maatregelen, en duurden deze episoden niet lange dan met huidige basale insulines.^5^ Deze studie van Svehlikova lijkt deze misperceptie over hypoglykemieën met wekelijkse insulines verder weg te nemen, maar kent mijns inziens een belangrijke valkuil. Zo werd het effect op hypoglykemieën "slechts" 24 uur geëvalueerd; de zorg is wat er de volgende dag(en) gebeurt. Het insuline-effect van overdosering met icodec zal langer aanhouden, terwijl het glargine-effect na 24 uur voorbij is. In de discussie na de presentatie werd duidelijk dat de T2D-patiënten in totaal 4 dagen werden vervolgd op de onderzoeksafdeling, met CGM en toediening van extra koolhydraten. Volgens de onderzoeker waren er in deze periode: 'Zeer weinig episoden van milde hypoglykemie, die allen gemakkelijk te corrigeren waren met orale koolhydraten.' Deze klinisch relevante bevindingen zijn (vooralsnog) niet officieel gerapporteerd. Robuust bewijs uit fase-III-studies, alsmede *real world* data, zullen waarschijnlijk nodig zijn om clinici (en patiënten) te overtuigen van de voordelen en veiligheid van wekelijkse insulines.

### Referenties

1. Russell-Jones D, Bode BW, De Block C, et al. Fast-Acting Insulin Aspart Improves Glycemic Control in Basal-Bolus Treatment for Type 1 Diabetes: Results of a 26-Week Multicenter, Active-Controlled, Treat-to-Target, Randomized, Parallel-Group Trial (onset 1). Diabetes Care 2017;40(7):943-950.
2. Mathieu C, Hollander P, Miranda-Palma B, et al. Efficacy and safety of insulin degludec in a flexible dosing regimen vs insulin glargine in patients with type 1 diabetes (BEGIN: Flex T1): a 26-week randomized, treat-to-target trial with a 26-week extension. J Clin Endocrinol Metab 2013;98(3):1154-62.
3. https://www.novonordisk.com/content/nncorp/global/en/news-and-media/news-and-ir-materials/news-details.html?id=118349
4. Bajaj HS, Bergenstal RM, Christoffersen A, et al. Switching to Once-Weekly Insulin Icodec Versus Once-Daily Insulin Glargine U100 in Type 2 Diabetes Inadequately Controlled on Daily Basal Insulin: A Phase 2 Randomized Controlled Trial. Diabetes Care 2021;44(7):1586-1594.
5. Rosenstock J, Del Prato S. Basal weekly insulins: the way of the future! Metabolism 2022; 126:154924.

*Review: Marcel Muskiet, arts in opleiding tot internist - fellow endocrinologie en vasculaire geneeskunde, Amsterdam UMC*

## 11. Oxyntomodulin Analog LY3305677 (LY) Improves Glycaemic Control and Weight Loss in Healthy Volunteers and Subjects with Type 2 Diabetes (T2D)

Charles Benson, Lai-San Tham, Yu Du, Sirel Gurbuz, Deborah A. Robins, Kieren J. Mather, Cheng Cai Tang, Melissa K. Thomas ***Indianapolis, USA; Singapore, Singapore***

### Background

LY is an acylated peptide analog of oxyntomodulin, a dual glucagon and GLP-1 receptor agonist. We conducted 2 randomized, double-blind, phase 1 multiple ascending subcutaneous dosing studies in healthy subjects (S1, NCT03325387) and in T2D patients (S2, NCT03928379) to evaluate safety, tolerability, pharmacokinetics, and pharmacodynamics. 

### Methods

In S1, 4 cohorts of healthy subjects received placebo or LY once-weekly (QW) for 4 weeks. In S2, T2D subjects received placebo or LY QW in 2 cohorts with doses escalated over 12 or 16 weeks. A total of 54 subjects completed S1 and 24 patients completed S2. 

### Results

The most common treatment-emergent adverse events in both studies (decreased appetite, nausea, and diarrhea) were mostly mild in severity, and no deaths or serious adverse events were reported. Pharmacokinetic results were consistent between healthy subjects and T2D patients and supported weekly dosing. In healthy subjects, fasting glucose levels were decreased from baseline with increasing LY doses, and reductions in mean body weight from baseline were generally seen with increasing doses, with the largest change observed on Day 29. In T2D subjects during weeks 12-16, preliminary results of mean HbA1c changes from baseline ranged from -1.56% to -2.16% in LY-treated vs. -0.43% to -0.70% in placebo subjects, and mean body weight changes from baseline ranged from -2.30 kg to -11.24 kg in LY-treated vs. -0.35 kg to -2.03 kg in placebo subjects. Fasting glucose, triglycerides, cholesterol, and glucagon levels were decreased from baseline with LY vs. placebo at weeks 12-16. 

### Conclusion

We conclude that LY was well tolerated in both studies, with a safety profile similar to selective GLP-1 receptor agonists. LY has a promising pharmacodynamic profile with potential for therapeutic benefit in T2D, obesity, or other metabolic diseases.

**Gecombineerd commentaar op 11&12 onder abstract 12.**

## 12. LY3437943 (LY), a Novel Triple GIP/GLP-1/Glucagon Receptor Agonist, Provides Glucose Lowering and Weight Loss in Patients with T2DM after 12 Weeks of Treatment

Shweta Urva, Mei Teng Loh, Tamer Coskun, Yu Du, Charles Benson, Corina Loghin, Axel Haupt, Zvonko Milicevic ***Indianapolis, USA; Singapore, Singapore; Zionsville, USA; Vienna, Austria***

### Background

Multi-receptor incretin agonists are being developed for several metabolic disorders. LY is an investigational triple agonist with potent activity on glucose-dependent insulinotropic polypeptide (GIP), glucagon-like polypeptide-1 (GLP-1), and glucagon receptors. LY was safely studied in a prior first-in-human study and pharmacokinetics properties supported once weekly dosing. The primary objective of this randomized, double-blind, placebo-controlled, Phase 1 proof-of-concept study was to assess the safety and tolerability of multiple ascending doses of LY in patients with type 2 diabetes (T2D). 

### Methods

Seventy-two patients were randomized (9:3:1) to 5 rising dose cohorts of subcutaneous LY, placebo, and dulaglutide 1.5 mg, respectively. Within cohort, dose-escalation was implemented at highest 2 cohorts. Vital signs, laboratory data and adverse events (AEs) were monitored to assess safety and tolerability. Efficacy was assessed by monitoring change in glycated hemoglobin (HbA1c) and body weight at week 12.

### Results

The most common treatment-emergent AEs were gastrointestinal (nausea and diarrhea), which were mostly mild in severity. By week 12, mean systolic and diastolic blood pressure decreased from baseline in LY compared to placebo group, while pulse and heart rate increased from baseline within most LY cohorts and dulaglutide, but not with placebo. By week 12, mean HbA1c decreased from baseline in all groups, with higher doses of LY showing statistically significant placebo-adjusted decreases of up to 1.56%. Except at the initial cohort, dose-dependent decreases in mean placebo-adjusted body weight of up to 8.96 kg were observed with LY. LY3437943 exhibits safety and tolerability profile similar to other incretins. 

### Conclusion

Promising glycaemic and body weight loss efficacy within this study highlights the potential for LY to provide additional benefit versus existing therapies in treatment of T2D and obesity.

*Funding: Eli Lilly and Company*

### Commentaar 11 & 12

De effectiefste behandelingen van obesitas en T2D zijn gericht op de darmen, waaronder chirurgische opties (bijvoorbeeld Roux-en-Y gastric bypass), die aanhoudend gewichtsverlies en diabetesremissie kunnen veroorzaken. Ook vanuit de farmacotherapie is er interesse in de darm als bron voor medicijnen. Sommige darmhormonen, waaronder GLP-1 en GIP, maar ook oxyntomoduline en PYY, zijn de laatste jaren belangrijke therapeutische doelen geworden. Farmaceuten ontwikkelen op dit moment moleculen die de complexe (endocriene) effecten van bariatrie nabootsen, met hoop op effectievere behandelingen voor obesitas, metabole ziekten en NAFLD.

De effectiviteit van GLP-1RA's is uitgebreid bekend, zowel als glucoseverlagend medicijn in T2D, en als gewichtsregulerend geneesmiddel bij obesitas (liraglutide [Saxenda], semaglutide [Wegovy]).^1,2^ Hun glucoseverlagende werking berust onder andere op glucose-afhankelijke stimulatie van de insulinesecretie, maar ook remming van glucagon en vertraging van de maagontlediging. Aangenomen wordt dat GLP-1RA's gewichtsverlies induceren door effecten op eetlustregulerende neuronen in de hypothalamus.

Een nieuwer - inmiddels beproefd en bewezen - concept is het agoneren van zowel de GLP-1- áls de GIP-receptor. Tirzepatide (Mounjaro) is hiervan de eerste in zijn klasse. In T2D heeft het toedienen van alleen GIP weinig/geen effect op de insulinesecretie, wat leidde tot het concept van diabetes als GIP-resistente conditie. Er is echter een verband tussen glucose en GIP-geïnduceerde insulinesecretie; normalisering van glucosespiegels kan GIP-resistentie (gedeeltelijk) herstellen. Daarnaast verhoogt GIP de glucagonspiegels tijdens hypoglykemieën, en stimuleert het de opname van vetzuren in vetweefsel. In tegenstelling tot GLP-1 heeft GIP geen invloed op de maaglediging, en lijkt er geen remming op te treden van voedselinname. De indrukwekkende effectiviteit van tirzepatide op HbA1c en gewicht in T2D was reeds gepubliceerd (SURPASS-studies).^3^ Tijdens de ADA Scientific Sessions 2022 kwamen daarbij de ongeëvenaarde gewichtsreducerende effecten in personen met obesitas (de SURMOUNT-1-studie), waarin tirzepatide qua effectiviteit bariatrische chirurgie benadert.^4^

Hoewel tirzepatide voor het grote publiek hét geneesmiddel was van ADA 2022, toonde farmaceut Eli Lilly data van nog twee andere zeer interessante moleculen; beiden in vroege fase van ontwikkeling, maar in potentie nog effectiever. Deze middelen betrekken, naast het incretineconcept, ook glucagonreceptoragonisme in hun werkingsmechanisme. Glucagon is bekend als hormoon dat de glucose-output door de lever verhoogt, waardoor glucosespiegels stijgen tijdens een hypoglykemie. Maar glucagon ook is erkend als gewichtsregulerend hormoon. Acute toediening van glucagon remt de voedselinname, induceert lipolyse en verhoogt het energieverbruik (mogelijk levergemedieerd via FGF21 en FXR). Ondanks de veelbelovende effecten van glucagon op lichaamsgewicht hebben de hyperglykemische effecten van glucagon de therapeutische ontwikkeling van glucagon-analogen voor obesitas of T2D verhinderd. Echter, gelijktijdige toediening van glucagon met GLP-1 resulteert in gewichtsverlies dat verder gaat dan wat met GLP-1 alleen kan worden bereikt, terwijl de glucoseverlagende effecten van GLP-1 behouden blijven. Deze bevindingen wekten interesse om unimoleculaire combinaties van glucagon en GLP-1 te ontwikkelen (bijvoorbeeld cotadutide). Een soortgelijk effect mag verwacht worden van analogen van het - minder bekende - darmhormoon oxyntomoduline, waarvan het werkingsmechanisme niet geheel bekend is, maar wat in elk geval zowel GLP-1- als glucagonreceptoren stimuleert. Oxyntomoduline-analoog LY3305677, inmiddels omgedoopt tot mazdutide, resulteerde in deze vroege-fase humane studie in indrukwekkende effecten op HbA1c en gewicht. Daarnaast is er LY3437943, een agonist van GIP-, GLP-1- en glucagonreceptoren; een project dat bekendstaat als "Triple-G". Conceptueel is het gebruik ervan drieledig: 1) GLP-1R-agonisme leidt tot gewichtsverlies en insulinesecretie, 2) glucagonreceptoragonisme activeert complementaire mechanismen voor gewichtsverlies (met name verhoging van energieverbruik), en 3) GIP-receptoragonisme buffert de glucagongemedieerde hepatische glucoseproductie via versterking van de insulinesecretie. Hoewel in de eerste plaats een veiligheidsstudie in T2D, is de effectiviteit op HbA1c en lichaamsgewicht van dit molecuul ook opvallend hoog. In tegenstelling tot mazdutide bevindt LY3437943 zich al in fase 2, zowel voor obesitas als T2D. De resultaten en relatief milde bijwerkingenprofielen zijn hoopvol, maar toxiciteit ligt mogelijk toch op de loer. Enkele jaren geleden heeft Novo Nordisk de ontwikkeling van hun duale agonist als tri-agonist stopgezet vanuit veiligheidsoverwegingen.^5^ Geen van de problemen die destijds werden gezien met Novo's GLP-1-/glucagonagonist zijn tot dusver opgedoken met mazdutide of LY3437943, maar alertheid zal noodzakelijk zijn in lopende vervolgonderzoeken.

### Referenties

1. Pi-Sunyer X, Astrup A, Fujioka K, et al. A Randomized, Controlled Trial of 3.0 mg of Liraglutide in Weight Management. N Engl J Med 2015;373(1):11-22.
2. Wilding JPH, Batterham RL, Calanna S, et al. Once-Weekly Semaglutide in Adults with Overweight or Obesity. N Engl J Med 2021;384(11):989-1002.
3. Moura FA, Scirica BM, Ruff CT. Tirzepatide for diabetes: on track to SURPASS current therapy. Nat Med 2022;28(3):450-451.
4. Jastreboff AM, Aronne LJ, Ahmad NN, et al. Tirzepatide Once Weekly for the Treatment of Obesity. N Engl J Med. 2022 Jun 4. doi: 10.1056/NEJMoa2206038. Online ahead of print.
5. https://www.evaluate.com/vantage/articles/interviews/novo-adds-weight-its-obesity-goals

*Review: Marcel Muskiet, arts in opleiding tot internist - fellow endocrinologie en vasculaire geneeskunde, Amsterdam UMC*

## 13. ADA Presidents' Select Abstract: Glycemic Outcomes over 12 Months in Very Young Children with the Omnipod 5 Automated Insulin Delivery (AID) System

Daniel Desalvo, Bruce W. Bode, Gregory P. Forlenza, Lori M. Laffel, Bruce A. Buckingham, Amy B. Criego, Melissa Schoelwer, Sarah A. Macleish, Jennifer Sherr, David W. Hansen, Trang T. Ly, Group Omnipod 5 In Preschoolers Study ***Houston, Atlanta, Aurora, Boston, Stanford, Minneapolis, Charlottesville, Cleveland, New Haven, Syracuse, Acton, USA***

### Background

The Omnipod 5 AID System is a tubeless hybrid closed-loop system with on-body operation and customizable glucose targets. 

### Methods

Safe and effective system use was demonstrated in children aged 2-5.9 years with type 1 diabetes (T1D) during a 3 month pivotal study. To evaluate durability of glycaemic benefit, we analyzed results from 9 months of an ongoing extension study, totalling 12 months of system use. In the pivotal study, participants used the system for 3 months at home, after 14 days of their standard therapy (ST, pump or multiple daily injections). They were then invited to participate in the extension study.

### Results

Safety endpoints were occurrence of severe hypoglycaemia (SH) and diabetic ketoacidosis (DKA). Glycaemic outcomes were A1C and percent time in ranges (TIR 70-180 mg/dL, TBR < 70 mg/dL, TAR > 180 mg/dL) during 3 month AID intervals compared with ST. All pivotal trial participants (n = 80), aged (mean ± SD) 4.7 ± 1.0 year with T1D duration 2.3 ± 1.1 year and total daily insulin of 14 ± 4U (range: 5.3-27U) at baseline, enrolled in the extension. Improved outcomes were observed for up to 12 months, including lower A1C and greater TIR during each AID interval compared with ST (all p < 0.05, Table). There were no episodes of DKA or SH in the 12 month study.

**Tabel 3.** Glycemic outcomes in very young children (N=80) aged 2-5.9 years at baseline/during standard therapy (ST) and during 3-month intervals for a total of 12 months of Omnipod 5 Automated Insulin Delivery (AID) System use Data are mean + SD or median [IQR] * Significant change from standard therapy as assessed by two-sided Wilcoxon signed rank or unadjusted paired t-test, p <0.05 ^1^AIC is measured at the end of each 3-month interval and was missing for 5 participants for Months 4-6 and 1 participant for Months 10-12 ^1^ One participant withdrew from the study during Months 10-12

**Measurement**	**Baseline/ST**	**AID Months 1-3** **(Pivotal study)**	**AID** **Months 4-6**	**AID** **Months 7-9**	**AID** **Months 10-12**^1^
**AIC,** %^1^	7.4 ± 1.0	6.9 ± 0.7*	7.0 ± 0.7*	7.1 ± 0.7*	6.9 ± 0.7*
**Meanglucose, **mg/dL	171 ± 31	157 ± 17*	157 ± 19*	158 ± 18*	159 ± 18*
**Sensor glucose percent time in ranges,** %					
**Time Below Range** (TBR), <70 mg/dL	2.19 [0.89, 4.68]	1.94 [1.18, 3.43]*	2.13 [1.23, 3.45]	2.38 [1.30, 3.22]	1.85 [1.16, 3.14]*
**Time In Range (TIR),** 70-180 mg/dL	57.2 ± 15.3	68.1 ± 9.0*	68.2 ± 9.7*	67.5 ± 9/3*	67.6 ± 9.6*
Time Above Range (TAR), >180 mg/dL	39.4 ± 16.7	29.5 ± 9.8*	29.2 ± 10.6*	29.8 ± 10.2*	30.0 ± 10.4'

### Conclusion

The safety and improved glycaemic outcomes from the initial 3 month pivotal study persisted for an additional 9 months, indicating the potential long-term benefit of the Omnipod 5 System in very young children with T1D.

*Funding: Insulet Corporation*

### Commentaar

De internationaal vastgestelde consensusdoelen voor glucoseregeling (Battelino 2019) zijn scherp en voor veel mensen alleen bereikbaar door het gebruik van automatische insulinetoediening (AID). Er zijn vijf zogenoemde 'hybrid closed-loop'-systemen beschikbaar, drie in Nederland, toegestaan voor gebruik > 6-7 jaar. De AID van Insulet, Omnipod 5, is gebruikt bij jonge kinderen (2-6 jaar oud, 20% 2-4 jaar). Op deze leeftijd is glucoseregeling om allerlei redenen extra lastig (lange nachten, infecties, wisselende intake, en dergelijke) maar des te belangrijker (neurocognitieve ontwikkelingsstoringen door hyper- en hypoglykemie). In deze 12 maanden multicenter (10) single-arm, poliklinische studie met 80 kinderen (15% gebruikte MDI, de rest al op een pomp; start HbA1c was laag met 57 mmol/l (7.4%)) maar daalde naar 6.9% met overeenkomstige verbetering TIR (57% naar 68%). Hypo's (TBR, time below range) bleef in beide groepen laag). Deze verbeteringen werden vrijwel al in 4-6 weken behaald en bleven stabiel over de 12 maanden. De grootste verbeteringen werd gezien tijdens de nachten. Hoewel we bij deze jonge kinderen niet de hoge TIR's en andere targets bereiken die bij volwassenen worden gezien (TIR 75-80%, hetgeen ook niet mag worden verwacht bij een algoritme dat niet specifiek voor deze leeftijdsgroep is ontwikkeld), is dit een belangrijke verbetering voor deze kwetsbare groep. De behandeling is veilig. Natuurlijk zijn grotere studies nodig, maar samen met de CAMaps AID opent de Omnipod 5 de weg naar gebruik van AID's en betere uitkomsten bij zeer jonge kinderen.

*Review: Henk-Jan Aanstoot, kinderarts-diabetoloog, Diabeter*

## 14. Effect of 24 Months of Optimised Glucose Control on Residual C-Peptide Secretion in Youth with New Onset Type 1 Diabetes (T1D)

Charlotte K. Boughton, Julia Ware, Janet M. Allen, Malgorzata E. Wilinska, Sara Hartnell, Ajay Thankamony, Tabitha Randell, Atrayee Ghatak, Rachel Besser, Daniela Elleri, Nicola Trevelyan, Fiona Campbell, Judy Sibayan, Ryan Bailey, Peter Calhoun, Gareth J. Dunseath, Roman Hovorka, Cloud Consortium***Cambridge, Nottingham, Liverpool, Oxford, Edinburgh, Southampton, Leeds, Swansea, United Kingdom; Tampa, USA***

### Background

We assessed whether improved glucose control with hybrid closed loop can preserve C-peptide secretion compared to standard insulin therapy in youth with T1D.

### Methods

In a multicentre, randomised, parallel trial, youth aged to < 17 years were randomised within 21 days of T1D diagnosis to hybrid closed loop using Cambridge algorithm (CL) or standard insulin therapy (control) for 24 months. Analysis was by intention to treat.

**Figuur 3.**
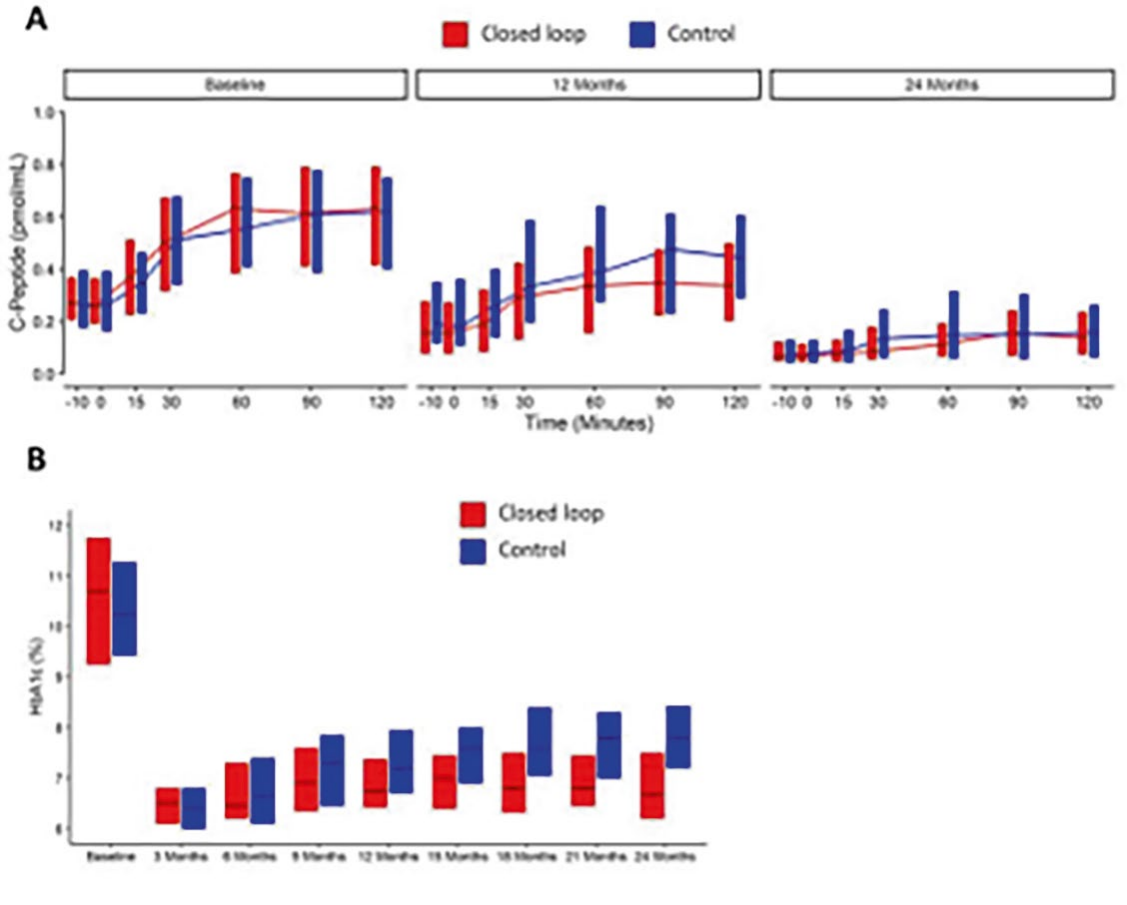
Panel A: Stimulated C-peptide at mixed meal tolerance test at baseline, 12 and 24 months. Horizontal bars in the boxes indicate the medians, and the bottom and top of each box represents the 25th and 75th percentules, respecteively.

### Results

We randomised 97 participants (mean ± SD age 12 ± 2 yrs), 51 to CL and 46 to control. There was no difference in C-peptide AUC at 12 months (primary endpoint) or 24 months between groups (geometric mean [95% CI] 12 months CL: 0.35 pmol/mL [0.27, 0.43] vs. control: 0.46 pmol/mL [0.33, 0.61]; mean adjusted difference -0. [-0.14 to 0.03]; p = 0. and 24 months CL: 0.17 pmol/mL [0.12, 0.23] vs. control: 0.25 pmol/mL [0.12, 0.39]; mean adjusted difference -0. [-0.14 to 0.05]; p = 0.25). Glycated haemoglobin was lower in the CL group by 4 mmol/mol [0.4%] (95% CI 0 to 8 mmol/mol [0.0 to 0.7%]) at 12 months, and mmol/mol [0.9%], (95% CI 7 to 15 mmol/mol [0.4 to 1.5%]; p < 0.001) at 24 months. Five severe hypoglycaemic events occurred in CL group (3 participants), and two in control group (2 participants); one DKA occurred in the CL group.

### Conclusion

In new onset T1D, optimising glucose control for 24 months does not appear to prevent the decline in residual C-peptide secretion.

*Funding: National Institute for Health Research EME Grant (14/23/09) Leona M & Harry B Helmsley Charitable Trust Grant (#2016PG-T1D046) Additional support for the artificial pancreas work is from National Institute for Health Research Cambridge Biomedical Research Centre, and Wellcome Strategic Award (100574/Z/12/Z). Medtronic and Dexcom supplied discounted CGM devices, sensors and details of communication protocol to facilitate real-time connectivity. Abbott Diabetes Care provided Libre Pro sensors.*

### Commentaar

Een intrigerende issue over de langetermijnuitkomsten is de vraag of behoud van c-peptide kan worden bereikt vanaf de diagnose, nu bekend is dat de mensen met T1D die lang (tot tientallen jaren) na de diagnose nog een kleine hoeveelheid insuline aanmaken (microsecretie c-peptide) een betere glucoseregeling, minder hypoglykemieën en minder ernstige (microvasculaire) complicaties hebben. Bij kinderen is een goede regeling des te belangrijker vanwege het negatieve effect van hypo's en hypers op de neurocognitieve ontwikkeling. Daarom werd in deze studie onderzocht of snelle introductie van automatische insulinetoediening (AID) resulteert in een beter behoud van insulineproductie (gemeten als c-peptidesecretie). In deze multicenter, gerandomiseerde parallel trial werden 97 kinderen (10-17 jaar)) binnen 21 dagen na diagnose gerandomiseerd voor AID (hybrid closed loop; CAMaps) of standaardbehandeling (CON). Primaire eindpunt was de c-peptidesecretie (AUC van de MMTT) na 12 en 24 maanden. Dat bleek niet het geval. De glucoseregeling was in de CAMaps-groep wel significant beter (HbA1c, TIR, TBR). De studie laat dan ook wel zien dat directe AID-therapie na diagnose toepasbaar en beter is bij kinderen. De vraag blijft open of c-peptidebehoud door een scherpe regeling bijdraagt. In andere presentaties werd opnieuw gewezen op een ander 'endotype' van T1D bij kinderen met veel agressievere auto-immuniteit. Verder zullen er naast de biologische (immunologische en bètacelspecifieke) factoren ook educatieve, psychologische en genetische factoren een rol kunnen spelen.

*Review: Henk-Jan Aanstoot, kinderarts-diabetoloog, Diabeter*

## 15. Multicenter MRI Assessment of the Pancreas in Type 1 Diabetes (MAP-T1D) Predicts Progression of Type 1 Diabetes

John Virostko, Jordan J. Wright, Jonathan M. Williams, Melissa A. Hilmes, Taylor M. Triolo, Hali C. Broncucia, Liping Du, Hakmook Kang, William E. Russell, Louis H. Philipson, Thomas Kay, Helen E. Thomas, Siri Atma W. Greeley, Andrea Steck, Alvin C. Powers, Daniel J. Moore

### Background

Pancreas size, as measured by MRI, is smaller at the onset of T1D and in individuals at risk for T1D. To determine whether pancreas MRI predicts T1D progression, we measured pancreas size and shape longitudinally in Type 1 Diabetes TrialNet Pathway to Prevention study participants. 

### Methods

To increase enrollment and expand this study across multiple TrialNet sites, we established the Multicenter Assessment of the Pancreas in T1D (MAP-T1D) group to develop harmonized MRI acquisition and image processing protocols and ensure the consistency of imaging results across sites. Using this standardized protocol (PMID: 34428220), we performed longitudinal pancreas MRI in 41 multiple autoantibody-positive individuals (mean age years old, range 8 - 45 years old, 49% female) at three TrialNet Clinical Centers. 

### Results

Average time between study entry and diabetes diagnosis for individuals who progressed to Stage 3 was 54 months while non-progressors were followed for a median time of 63 months. Five study participants who developed Stage 3 T1D during follow up had a smaller pancreas (29.5 ± 9.0 ml vs. 54.8 ± 22.5 ml, p = 0.02) and smaller pancreas volume normalized by body weight (0.61 ± 0.18 ml/kg vs. 0.85 ± 0.23 ml, p = 0.03) at study entry compared with those who did not progress to Stage 3. We found that pancreas volume was stable over time, with no significant increase or decrease up to four years after the initial MRI (total of 123 MRIs). There was no significant change in pancreas size in the five individuals who progressed to Stage 3 T1D. The shape of the pancreas at study onset was also different in the five individuals who progressed to Stage 3 T1D compared with non-progressors, with larger surface area to volume ratio (p = 0.02), and shorter principal axes (p = 0.05, shortest axis; p = 0.02, second shortest axis). 

### Conclusion

These data suggest that small pancreas size and altered shape predicts progression from Stage 2 to Stage 3 T1D.

*Funding: NIDDK (R03DK129979, U24DK097771, UC4 DK106993, DK020593); JDRF International (3-SRA-2015-102-M-B, 3-SRA-2019-759-M-B); Thomas J. Beatson, Jr. Foundation (2021-003)*

### Commentaar

Type 1-diabetes wordt veroorzaakt door een bètacelprobleem dat een auto-immuunreactie kan uitlokken. De vermindering (absoluut en functioneel) van bètacellen resulteert ook in veranderingen in het exocriene weefsel. De alvleesklier wordt kleiner bij T1D (hetgeen soms gepaard gaat met exocriene disfunctie). Dit "pancreasverkleiningsproces" is al voor de diagnose bij mensen die onderweg zijn naar T1D (gedefinieerd door de aanwezigheid van auto-antistoffen (stadium 1) en eventueel plus afwijkend glucose (stadium 2)) te meten. Dit MRI-onderzoek wil het longitudinale beloop van dit proces vastleggen als eventueel merker van op handen zijnde T1D. Na standaardisatie van de *pancreas volume index* (PVI) werd gemeten bij mensen in stadium 1(n = 16) of stadium 2 (n = 21) en 'progressors' naar stadium 3 (n = 7; klinische verschijnselen en diagnose). Er werd een afname van PVI gezien over de vijf jaar observatieperiode tussen deze stadia (ROC-AUC 0.838 (84%) p = 0.005). T1D was het beste te voorspellen bij hen die een PVI onder het normale gemiddelde hadden, en was vooral uit de dikte van de pancreas (en niet de lengte) te voorspellen. Dit zal (25 minuten per MRI) nog geen 'standaardmethode' worden, maar nu wereldwijd grote predictiestudies lopen in zowel de populatie als bij familieleden van mensen met T1D, kan deze MRI-methode meehelpen om de predictiemodellen te verfijnen. Ook zal het (naast meting van genetisch risico en/of de aanwezigheid van auto-antistoffen) helpen om de heterogeniteit van de ziekte in beeld te krijgen als 'endotypes' van op handen zijnde T1D. Dat is een voorwaarde voor verdere predictie- en precisietherapie van T1D. Ten slotte wijst dit onderzoek uit om bij klachten te denken aan een kleiner wordende pancreas en mogelijke exocriene disfunctie.

*Review: Henk-Jan Aanstoot, kinderarts-diabetoloog, Diabeter*

## 16. ADA Presidents' Select Abstract: Impact of Overnight Blood Glucose on Next-Day Functioning in T1D

Elizabeth Pyatak, Donna Spruijt-Metz, Stefan Schneider, Jill P. Crandall, Anne L. Peters, Haomiao Jin, Shivani Agarwal, Loree T. Pham, Aina Ali, Claire J. Hoogendoorn, Gladys Crespo-Ramos, Heidy Mendez-Rodriguez, Pey-Jiuan Lee, Valerie F. Ruelas, Rose Basile, Jeffrey S. Gonzalez ***Los Angeles, Bronx, New York, USA***

### Background

While people with T1D report that blood glucose (BG) fluctuations affect their day-to-day functioning, these relationships are poorly understood. Using continuous glucose monitoring (CGM), accelerometry, and momentary surveys and cognitive tasks, we sought to understand how various BG metrics during sleeping hours impact functioning the following day.

### Methods

Participants wore a blinded CGM and accelerometer for 10-14 days, during which they completed surveys and cognitive tasks 5-6 times per day. Using dynamic structural equation modeling, we evaluated the within-person impact of overnight BG on the following day's functioning, while controlling for the prior day's functioning. BG variables included % time < 70 mg/dL, % time > 250 mg/dL, and coefficient of variation (CV). Functioning variables included cognitive measures of sustained attention and perceptual speed; daily step count; and self-reported fatigue, task performance, and net activity demand.

### Results

Among 127 adults with T1D (41 ± 15 yrs, 46% male, 39% Latino, 35% White, 10% Black, 4% Asian, 12% multi-ethnic/other), all three BG variables predicted changes in next day functioning. More time < 70 predicted poorer sustained attention (standardized b = -0.06), while more time > 250 predicted more fatigue (0.09) and lower step count (-0.12) (all p < 0.05). Higher CV had the broadest impact, predicting poorer sustained attention (-0.06), more fatigue (0.05), and less engagement in demanding activities (-0.05) (all p < 0.05).

### Conclusion

Our findings indicate that overnight BG impacts cognition, physical activity, fatigue, and activity engagement the following day. While effect sizes are small, the cumulative impact of these decrements in function should be considered in the context of a life-long disorder. The consistent findings among diverse outcomes, including both objective and self-report measures, strengthen confidence in the overall conclusion that overnight BG has wide-ranging implications for functioning in adults with T1D.

*Funding: National Institutes of Health (1R01DK121298-01)*

### Commentaar

Kwaliteit van leven (KvL) is een belangrijke parameter naast de uitkomsten van glucoseregeling (HbA1c, TIR en andere *glucometrics*). Het meten van aan KvL gerelateerde onderdelen kan beïnvloed worden door zogenoemde *within-person dynamics*, zoals de directe invloed van de glucosewaarde op het functioneren van iemand met T1D. De onderzoekers wilden daarom de vraag beantwoorden of de nachtelijke glucosewaardes en hun variabiliteit voorspellend zijn voor zowel het zelfgerapporteerde als het objectief gemeten functioneren van de volgende dag. Ze deden dat door meting van glucosewaardes (geblindeerde CGM LibrePro), activiteiten (accelerometrie) en een *ecological momentary assessment* (EMA) bestaande uit 5-6 maal per dag (op smartphone) een *self-reported survey* (bijvoorbeeld: 'Hoe gestrest voel je je nu?') plus een cognitieve test (reactiesnelheid en aandacht). Er deden 127 mensen mee (41.5 jaar oud, 54% vrouw, 29% op AID/hybrid-closed loop, 39% op CGM en 30% geen CGM; gemiddelde TIR 58%). De nachtelijke glucosewaarde voorspelde de uitkomst van de EMA's. De glucosevariabiliteit had het meeste effect op het functioneren: hoe hoger de CV, des te minder aandacht, meer vermoeidheid en minder activiteiten (accelerometer). De auteurs pleiten voor meer aandacht voor de glucosevariabiliteit (bijvoorbeeld door betere CV bij iets hogere glucoses). In een subanalyse bevestigden ze de relatie tussen de glucosewaarde en *self-efficacy, diabetes distress* en de impact daarvan op zelfzorg. Hoewel dit nog kleine onderzoeken betreft, is het belangrijk te realiseren dat − naast het HbA1c − ook het 'Hé-hoe-gaat-het-ermee' kan worden gemeten, maar dit ook wordt beïnvloed door vele factoren.

*Review: Henk-Jan Aanstoot, kinderarts-diabetoloog, Diabeter*

DIT CONGRESVERSLAG IS MEDE MOGELIJK GEMAAKT DOOR NOVO NORDISK.

NOVO NORDISK HEEFT GEEN INVLOED GEHAD OP DE INHOUD VAN DE BIJDRAGEN.

